# Assessing undergraduate mentoring competency in a research-intensive Hispanic serving institution: A revalidation

**DOI:** 10.1371/journal.pone.0350417

**Published:** 2026-06-25

**Authors:** Rafael Aguilera, Robert McCreary, Jaime Gutierrez Portillo, Amy E. Wagler

**Affiliations:** 1 Research Evaluation and Assessment Services, The University of Texas at El Paso, El Paso, Texas, United States of America; 2 Department of Mathematical Sciences, The University of Texas at El Paso, El Paso, Texas, United States of America; 3 Research Cores Program, New Mexico State University, Las Cruces, New Mexico, United States of America; Lamar University, UNITED STATES OF AMERICA

## Abstract

Mentoring is a critical factor in fostering persistence and professional identity among undergraduate students from underrepresented minority (URM) backgrounds in STEM. However, the systematic assessment of mentor effectiveness, particularly within diverse institutional contexts, remains a challenge. The Mentoring Competency Assessment (MCA), though widely used, was originally validated on a homogeneous sample and subsequent revisions (MCA-21) relied on traditional psychometric methods (PCA/CFA), leaving concerns about its applicability and complexity in highly diverse settings. This study addressed the need for a culturally relevant and psychometrically rigorous assessment by examining the factor structure of the MCA-21 among research mentors (*N* = 323) at a Hispanic-Serving Institution (HSI). We utilized Exploratory Graph Analysis (EGA), a network-based method that identifies latent structures without imposing rigid model assumptions, to assess how mentoring competencies cluster in this unique context. EGA results revealed a more parsimonious three-factor structure, significantly differing from the original six-factor model. This abbreviated structure suggests that effective mentorship at an HSI may be best captured by clusters focusing on (1) Research Skill Development, (2) Identity and Belonging, and (3) Career Alignment. These findings indicate that the structure of mentoring competencies is context-dependent, potentially integrating culturally responsive practices into core domains. The resulting three-factor structure provides a validated, context-specific, and less burdensome instrument for HSI settings (CLI = 0.951). This refinement enhances the utility of the MCA for evaluating mentor effectiveness, improving the precision of mentor training programs, and ultimately fostering equitable professional development for URM students in diverse academic environments.

## Introduction

Mentoring plays a critical role in the development of research skills, professional identity, and career trajectories of undergraduate students, particularly those from underrepresented backgrounds in STEM fields [1]. Effective mentoring enhances research self-efficacy, fosters a sense of belonging, and supports the retention of students in research careers, making it a key mechanism for broadening participation in science. Tailored mentoring approaches for minoritized students are crucial for addressing systemic barriers and supporting the professional development of underrepresented minority (URM) students. For example, Latino students in higher education face systemic barriers that hinder their academic success, including financial constraints that limit access to essential resources [2], a lack of culturally responsive mentorship opportunities that contribute to feelings of isolation [3], and structural inequities in academic preparation and institutional support that affect college readiness and persistence [4]. Addressing these challenges through targeted mentoring and institutional interventions is critical for fostering equitable educational and professional outcomes.

Still, despite the recognized importance of mentoring, there has been a historical lack of structured, validated assessments to measure mentoring competencies. Many institutions rely on informal feedback or self-reported assessments, that may not accurately capture mentor effectiveness or provide actionable insights for improvement. Developing robust evaluation tools for mentoring is essential for advancing evidence-based mentor training and ensuring that mentoring relationships contribute meaningfully to student success.

### The mentoring competency assessment (MCA)

To address the need for a structured approach to evaluating mentoring competencies, Fleming and colleagues (2013) developed the Mentoring Competency Assessment (MCA), a 26-item instrument designed to assess six key areas of mentoring proficiency: (1) maintaining effective communication, (2) aligning expectations, (3) assessing understanding, (4) addressing diversity, (5) fostering independence, and (6) promoting professional development. The MCA was initially validated in a national study involving mentor-mentee pairs from clinical and translational science programs, demonstrating strong reliability and validity as a tool for assessing mentor effectiveness.

Since its initial development, the MCA has been widely adopted; however, its applicability to more diverse institutional and disciplinary contexts has not been thoroughly examined. The original validation was based on a relatively homogeneous sample of predominantly senior, white male faculty in biomedical fields [5]. This lack of diversity limited the instrument’s generalizability. A subsequent revalidation by Hyun and colleagues (2022) sought to refine the MCA using a larger, more diverse sample, resulting in a revised 21-item version (MCA-21) that retained the six key competency domains. Despite these improvements, traditional validation methods such as Principal Component Analysis (PCA) and Confirmatory Factor Analysis (CFA) were primarily used, leaving room for further exploration using modern psychometric techniques.

Mentorship in research settings is widely recognized as a key factor in fostering academic and professional success, particularly for students pursuing STEM careers. Several theoretical frameworks help explain how mentoring contributes to mentee development by shaping career trajectories, strengthening professional identity, and enhancing self-efficacy. Social Cognitive Career Theory [6] and Self-Determination Theory [7] both emphasize the role of mentorship in reinforcing competence, autonomy, and motivation, ultimately guiding mentees toward sustained career commitment. From a sociocultural perspective, Communities of Practice Theory [8] and Cultural Mismatch Theory [9] highlight how mentorship helps mentees navigate disciplinary norms and institutional barriers, particularly for those from underrepresented backgrounds. Meanwhile, the Model of Academic Socialization [10] underscores the role of mentorship in integrating mentees into scholarly communities by fostering socialization into the values, skills, and expectations of academic and research careers. Together, these frameworks illustrate how effective mentorship goes beyond skill development, serving as a mechanism for identity formation, persistence in STEM, and long-term professional success.

Mentoring has well-documented benefits for mentees, particularly those from underrepresented backgrounds in STEM. Studies have shown that mentorship can enhance science identity, improve research self-efficacy, and increase persistence in STEM careers [11,12]. URM students often face systemic barriers such as imposter syndrome, financial constraints, and a lack of role models in academia [4]. Culturally responsive mentorship, which involves mentors acknowledging and integrating students’ backgrounds into their mentoring approach, has been shown to significantly enhance URM students’ research engagement and retention [13].

Formalized mentoring interventions have been shown to significantly enhance the academic and research trajectories of URM students in STEM. Research indicates that structured mentorship programs contribute to increased graduate school enrollment, higher research productivity, and improved persistence in STEM fields by providing mentees with targeted training, professional development opportunities, and socialization into the research community [14,15]. These programs often incorporate evidence-based practices such as tiered mentoring structures, research apprenticeships, and culturally responsive mentoring approaches to address barriers faced by URM students in higher education. Although these interventions are beneficial, there remains a critical gap in the systematic assessment of mentoring competencies across different institutional contexts. Many mentoring programs operate without validated evaluation frameworks, making it difficult to measure their effectiveness and refine mentorship practices based on empirical evidence. Addressing this gap requires robust mentoring assessments that capture key competencies and account for contextual differences in mentorship experiences, particularly at institutions serving diverse student populations.

Many of the various mentoring assessment tools that currently exist are contextually limited or lack psychometric rigor. For example, the Mentorship Effectiveness Scale (MES) [16] focuses primarily on mentee satisfaction rather than mentor skill development. Similarly, the Wisconsin Mentoring Seminar Evaluation [17] and the Mentor Role Instrument (MRI) [18] provide valuable insights into mentor-mentee interactions but are not widely validated across diverse disciplines or institution types. One of the most comprehensive tools, the MCA, was developed by Fleming et al. [19] and has been widely adopted in clinical and translational research mentoring contexts. However, the original validation sample was limited in diversity, comprised primarily of senior faculty in biomedical sciences, which raises concerns about its generalizability to broader research settings, including Minority Serving Institutions (MSIs), more broadly, and non-biomedical disciplines.

### The need for further validation

Despite efforts to refine the MCA, additional validation within Hispanic-Serving Institutions (HSIs) and other Minority-Serving Institutions (MSIs) remains necessary. Mentoring practices at HSIs, as well as at other MSIs such as Historically Black Colleges and Universities (HBCUs), may differ substantially from those at predominantly White institutions due to variations in institutional culture, student demographics, and resource availability [20]. HSIs, in particular, serve a high proportion of first-generation, bilingual, and socioeconomically disadvantaged students, which shapes both the structure and function of mentoring relationships and the types of support needed to foster persistence in research careers [21]. As a result, mentorship in HSI contexts often integrates family engagement, cultural responsiveness, and collectivist support networks, whereas mentoring models at predominantly White institutions more frequently emphasize individualized academic skill-building [13]. Prior research further indicates that Latino students benefit from mentorship approaches that explicitly acknowledge cultural background and emphasize familismo, community-based learning, and bicultural navigation [14,22], with culturally responsive mentoring in HSIs associated with higher research self-efficacy, stronger science identity, and increased graduate school enrollment [23]. However, systematic evaluation of mentoring competencies within HSI settings remains limited, creating an assessment gap in determining whether existing instruments such as the MCA adequately capture these context-specific mentoring practices. Expanding validation of the MCA in HSI contexts is therefore critical to ensure construct relevance across institutional types and to support the development of evidence-based mentor training interventions tailored to underrepresented student populations.

Traditional psychometric validation techniques, such as PCA and CFA, rely on predefined factor structures and linear assumptions that may oversimplify complex, multidimensional constructs such as mentoring effectiveness. EGA is particularly useful in mentoring research because mentoring relationships are inherently complex, dynamic, and context dependent [24]. Exploratory Graph Analysis (EGA) [25] is a contemporary, data-driven approach that uses network analysis to identify linear and non-relationships and the natural clustering of items, providing a more nuanced understanding of underlying factor structures without imposing rigid model assumptions. Given that previous revalidations relied on traditional methods, applying EGA can lead to a more contextually relevant and parsimonious assessment tool for institutions serving diverse student populations. The present study addresses these critical gaps by applying Exploratory Graph Analysis (EGA) to assess the factor structure of the MCA using a dataset of research mentors at a Hispanic-Serving Institution (HSI). This revalidation effort aims to provide a more contextually relevant and parsimonious assessment tool for institutions serving diverse student populations. Specifically, this study is guided by the following research questions:

1. What is the latent factor structure of the MCA when administered to research mentors at a HSI, as determined by EGA?
2. How does the factor structure identified by EGA compare to the original six-factor model [19] and previously revalidated structures (e.g., [5])?
3. What are the implications of the resulting refined MCA structure for developing evidence-based mentor training programs tailored to support diverse student populations at HSIs?

## Methods

### Setting

El Paso, Texas, and Ciudad Juarez, Mexico, are neighboring cities located along the U.S.-Mexico border in western Texas. Nearly 75% of households in this area are bilingual in Spanish and English, making it one of the largest bicultural and bilingual metropolitan regions in North America. El Paso itself has an 83% Hispanic population [26]. Despite this cultural richness, the area faces significant poverty, with 21% of families living below the poverty line, compared to 14% in Texas and 11.5% nationwide. The median household income in El Paso County is $55,417, which is notably lower than the Texas median of $73,035 and the U.S. median of $75,149. Additionally, only 25% of El Paso County residents have a bachelor’s degree or higher, compared to 32.3% in Texas and 34.3% nationally.

Similarly, the University of Texas at El Paso (UTEP) has a student population that is approximately 84.23% Hispanic, making it the first national research-intensive university serving a 21st-century underrepresented demographic. Nearly half of UTEP’s over 23,000 students are first-generation college students (Texas Monthly, 2023; UTEP, n.d.). These factors underscore the community’s need for effective educational interventions. Successful interventions in this community are likely to benefit the broader academic community, as they address the needs of both minoritized (e.g., racial/ethnic minorities) and marginalized (e.g., low socioeconomic status) populations. Therefore, interventions that prove effective in this environment can be expected to work well in other settings.

### Measures

The Mentor Competency Assessment (MCA) [19] is a 26-item measure developed to assess core mentoring competencies. Items are rated on a 7-point Likert-type scale ranging from 1 (“not at all skilled”) to 7 (“extremely skilled”) by both mentors and mentees. It is important to note that the MCA survey was administered to all participants exclusively in English. This is a potential limitation given the HSI’s location in a large, bicultural, and bilingual metropolitan region. The six domains include: maintaining effective communication, aligning expectations, assessing understanding, addressing diversity, fostering independence, and promoting professional development (Please see Table 2 in Appendix for a full list of factors and items). The full 26-item MCA measure was used for this study to enable direct comparisons with prior validation work and to further explore its dimensionality using modern psychometric tools (i.e., EGA).

### Data preparation

The initial dataset comprised 367 MCA survey responses submitted by mentors, with some mentors submitting multiple responses within a single year due to assessing multiple mentees or submitting duplicate survey entries. A convenience sampling strategy was employed, as all mentors participating in undergraduate mentored research experiences between 2017 and 2024 were invited to respond to the survey. To prepare the dataset for analysis, we first retained only one survey response per mentor per year by grouping responses by unique mentor ID and calendar year. At this step, surveys with the most complete data were selected, reducing the dataset to 363 responses. Next, missingness was examined among MCA items, revealing at most 7.2% missing data. Pairwise deletion was implemented where possible; otherwise, listwise deletion was used reducing the original 363–323. However, 58.5% of data indicating ethnicity of the mentor was missing. Of the non-missing values, 29.5% were Hispanic and the remaining non-Hispanic. The final analytical sample size of *N* = 323 was later supported by a post-hoc Monte Carlo power analysis, which confirmed 100% power to detect all factor loadings for the resulting factor structure.

### Statistical approach

Exploratory graph analysis and factor analysis are both methods used in statistics to explore relationships and patterns within data, but they differ in their approaches. FA and PCA approaches there are strict assumptions made about the relationships between items (aka linearity) and latent variables are often restricted to be independent. This is not feasible in many applied settings. Instead, EGA provides a more flexible and realistic approach to modeling the structure and characteristics of constructs used in research training but maintains a strong connection to the fundamental objectives of the PCA and FA approaches. In particular, if a latent variable model accurately represents the structure of a scale, there is evidence that these latent scores or factors are similar to the interconnected clusters of indicators within a network model. In a network model, edges signify partial correlation coefficients between variables after considering all other variables. Since two indicators influenced by the same latent variable cannot become independent after accounting for observed variables, the strength of the edge between them shouldn’t be zero. Mathematically, network models can be demonstrated to be equivalent to latent variable models under specific conditions, wherein each latent variable corresponds to a unidimensional structure.

### Sub-scale identification in EGA

To detect community structure or EGA sub-scales, we employ community detection algorithms, a type of clustering algorithm that provides multiple perspectives on the potential community configurations (sub-scales) among the MCA items. This is especially crucial when clustering outcomes are theoretically specified in advance, as in the present study, to identify which algorithm aligns best with the hypothesized structure [27]. In this study, we examined three preeminent community detection algorithms to identify sub-scales in the MCA: the Walktrap, Leiden, and Louvain algorithms [19]. The resulting community structures were assessed for any problematic features, such as poorly connected subnetworks, but no connectivity issues were observed.

## Results

The final analyzed sample consisted of N = 323 mentors participating in undergraduate mentored research experiences between 2017 and 2024, with the highest number of participants from 2019 (n = 83, 23.1%) and 2018 (n = 74, 20.6%). As shown in Table A1 in the Appendix, the mentors represented a range of academic ranks. The most frequent ranks were Associate Professor (114, 32.4%), Professor (106, 30.1%), and Assistant Professor (85, 24.1%). A smaller proportion consisted of participants in training or non-faculty roles, including PhD Students (14, 4.0%), Graduate Students (13, 3.7%), and Post-doctoral researchers (10, 2.8%). Regarding gender identification based on the available data, 53.1% (43) identified as Male, 45.7% (37) identified as Female, and 1.2% (1) identified as Other.

### Modified parallel analysis

The modified parallel analysis supported a five-factor solution. Figure A1 in the Appendix provides a visual summary of the eigen analysis. Eigenvalues from the principal axis factor analysis were 15.012, 0.734, 0.624, 0.441, and 0.332 for the first five factors, with subsequent eigenvalues turning negative (−0.015 to −0.356). The 99th percentile of simulated eigenvalues for the first factor (0.595) is much smaller than the first observed eigenvalue (15.012), reinforcing the five-factor solution. The five factors explained 65.94% of the total variance (based on 26 items), with individual contributions of 57.74%, 2.82%, 2.40%, 1.70%, and 1.28%. Relative to the total common variance (positive eigenvalues only), the factors accounted for 84.36%, 4.13%, 3.51%, 2.48%, and 1.87%, respectively, cumulatively explaining 96.34% of the common variance. Further analysis of eigenvalue ratios highlighted a highly dominant first factor, with a ratio of 20.444 when compared to the second eigenvalue. Subsequent eigenvalue ratios (second to third, third to fourth, and fourth to fifth) were 1.177, 1.415, and 1.328, respectively, suggesting a more gradual decline in the variance accounted for by these later factors.

### Bootstrap exploratory graph analysis

We applied EGA using the EGAnet package [28] in R [29] to explore the underlying dimensionality of the MCA and identify a parsimonious factor structure. We initially conducted bootstrap EGA on all 26 MCA items to determine the most stable community structure. Multiple community detection algorithms were compared, including Walktrap, Louvain, and Leiden. However, the Walktrap algorithm was ultimately deemed unsuitable for our data because it prioritizes random walks through the network without adequately accounting for item-level associations relevant to psychological constructs. Instead, we selected a community detection algorithm that demonstrated greater sensitivity to the underlying covariance structure of the data, yielding more interpretable and substantively meaningful results.

We used iterative bootstrap EGA combined with item stability analysis to refine the MCA structure. This approach allowed us to identify items that consistently failed to cluster reliably across bootstrapped samples. Items with low item stability (<0.50) were considered for removal. Through successive iterations, we dropped poorly performing items to improve the clarity and parsimony of the factor solution while preserving the conceptual integrity of the instrument. This iterative refinement process culminated in a final EGA model composed of fewer items, each exhibiting high structural consistency and clear loading onto well-defined factors. The resulting solution was more stable and interpretable, aligning well with theoretical expectations of the MCA’s core competency domains.

**Fig 1:**
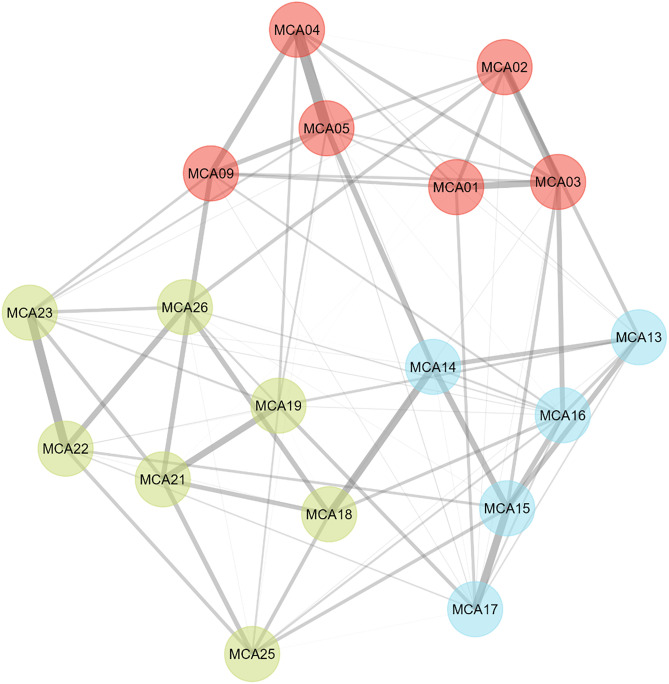
Network Plot of GLASSO EGA with Leiden algorithm (18-item).

Two stable factor configurations emerged from the bootstrap EGA analysis. One solution, identified by both the Leiden and Louvain algorithms, revealed a three-factor structure comprising 19 items (see Fig 1). All items in this configuration demonstrated stability values of 0.50 or greater across bootstrap samples, indicating robust item placement within their designated factors. The convergence of both algorithms on this structure suggests particular reliability of this solution. The Leiden algorithm additionally identified a more parsimonious three-factor solution containing 18 items. This structure retained all the stability criteria while offering a slightly more condensed version of the scale. Both solutions represent a significant departure from the originally hypothesized six-factor structure, suggesting a simpler underlying configuration of mentoring competencies in this sample. The convergence of both community detection algorithms on the 19-item solution, coupled with the availability of an 18-item alternative, suggests that mentoring competencies, as measured by the MCA, can be effectively represented by three core dimensions rather than the originally proposed six.

### Confirmatory factor analysis

Confirmatory factor analyses were conducted to evaluate the goodness-of-fit of four competing factor structures for the 26-item MCA: the original Fleming 6-factor, 26-item model; the revised Hyun 6-factor, 21-item model; a data-driven Louvain 3-factor, 19-item model; and a data-driven Leiden 3-factor, 18-item model. Model fit was assessed using the scaled chi-square statistic, robust CFI, robust TLI, robust RMSEA (and its 90% CI), and SRMR, as recommended for WLSMV estimation with ordinal data. Examination of the approximate fit indices revealed variations in fit quality across the models. The Fleming and Hyun models demonstrated poor fit, with Robust CFI and TLI values well below the acceptable threshold of 0.90 and Robust RMSEA values above 0.10. The Louvain and Leiden models showed improved fit, approaching or meeting acceptable criteria for some indices.

The Fleming model (6-factor, 26-item) showed less than ideal robust fit indices (Robust CFI = 0.83, Robust TLI = 0.80). The Robust RMSEA was 0.12 (90% CI: 0.12, 0.13), indicating poor fit according to common guidelines (RMSEA > 0.10), and the SRMR was acceptable at 0.04. The Hyun model (6-factor, 21-item) exhibited the poorest robust fit among the four models. Its Robust CFI (0.82) and Robust TLI (0.78) were the lowest, and its Robust RMSEA was the highest at 0.14 (90% CI: 0.14, 0.15), clearly indicating poor model fit. The SRMR was acceptable (0.05). See Table 1 for all details regarding comparative model fits.

**Table 1:** CFA Fit Indices.

	*Fleming* *(6-factor, 26-item)*	*Hyun* *(6-factor, 21-item)*	*Louvain* *(3-factor, 19-item)*	*Leiden* *(3-factor, 18-item)*
Scaled χ2	1134.34	1085.32	578.63	540.29
Scaled df	284	174	149	132
Scaled p-value	<.001	<.001	<.001	<.001
Robust CFI	0.83	0.82	0.90	0.91
Robust TLI	0.8	0.78	0.89	0.89
Robust RMSEA	0.12 [0.12, 0.13]	0.14 [0.14, 0.15]	0.10 [0.10, 0.11]	0.11 [0.10, 0.11]
SRMR	0.04	0.05	0.04	0.04

Note. CFI = Comparative Fit Index; TLI = Tucker-Lewis Index; RMSEA = Root Mean Square Error of Approximation; SRMR = Standardized Root Mean Square Residual. Scaled and Robust indices are reported as appropriate for the WLSMV estimator.

The Louvain model (3-factor, 19-item) demonstrated notably better robust fit compared to the Fleming and Hyun models. The Robust CFI was 0.90 and the Robust TLI was 0.89, meeting or approaching the conventional threshold of 0.90 for good fit. The Robust RMSEA was 0.10 (90% CI: 0.10, 0.11), which is at the upper limit of acceptable fit. The SRMR was good at 0.04. The Leiden model (3-factor, 18-item) showed the most favorable robust fit indices. Its Robust CFI was 0.91, exceeding the 0.90 threshold, and its Robust TLI was 0.89. The Robust RMSEA was 0.11 (90% CI: 0.10, 0.11), similar to the Louvain model and within or at the upper limit of acceptable fit. The SRMR was also good at 0.04.

Comparing the two better-fitting models directly, the Leiden model displayed a slightly more favorable CFI value (0.91) compared to the Louvain model (0.90). In contrast, the Louvain model had a slightly better RMSEA (0.10) compared to the Leiden model (0.11). The TLI and SRMR values were identical at 0.89 and 0.04, respectively). The differences in CFI (ΔCFI = 0.01) and RMSEA (ΔRMSEA = 0.01) are minimal and fall within the range often considered negligible for practical purposes, even if applying standard cutoffs cautiously. Given the slightly higher CFI, the Leiden (3-factor, 18-item) model was marginally preferred as the best statistical representation of the MCA factor structure in this sample, although the Louvain model represents a very closely competing alternative.

Standardized parameter estimates for the selected Leiden (3-factor, 18-item) model are presented in Table 2 alongside the other CFA models. For the Leiden model, all items exhibited statistically significant and substantial loadings on their designated factors, with standardized loadings (λ) ranging from 0.725 to 0.932. All loadings exceeded.70, indicating strong relationships between the items and their respective latent constructs and providing support for the convergent validity of the measurement model. The latent factors themselves were strongly positively correlated, with inter-factor correlations (Φ) ranging from 0.832 to 0.890. These high correlations suggest that the factors represent distinct but closely related aspects of mentor competency, providing evidence for discriminant validity while acknowledging the strong interconnectedness of the competencies.

### Three-factor model for mentoring competencies

The final factor solution selected for this study was the 18-item, 3-factor model identified using the Leiden community detection algorithm. Fig 1 provides a visual depiction of this model and Table 1 and 2 provide fit information. Additionally, Figures A2 and A3 in the Appendix provide information about the stability of the Louvain and Leiden models for each item as computed via the bootstrap simulation and Figure A4 provides the alternate structure implied by the Louvain algorithm for community detection. This model retained high item stability across bootstrap samples and offered a conceptually coherent, parsimonious structure that aligns with core mentoring competencies while improving interpretability compared to the original six-factor framework. A post-hoc power analysis conducted for this model using Monte Carlo simulation. To address the potential circularity of using sample estimates as population parameters and to account for ceiling effects found in the data, the power analysis included a sensitivity analysis wherein population parameters were weakened. Specifically, observed factor loadings were attenuated by 50%, and factor correlations were constrained to 0.50, simulating a conservative condition with weaker effects and lower collinearity than observed in the empirical data. The simulation, performed using the *simsem* package in R [30] with 1,000 replications, assessed the proportion of datasets in which the factor loadings were statistically significant. Even with reduced population loadings, the statistical power to detect every factor loading was 100%.

#### Factor 1: Maintaining effective communication.

This factor retained five of the six items from the original *Maintaining Effective Communication* factor, including active listening, providing constructive feedback, building trust, accommodating communication styles, and employing strategies to enhance communication. Additionally, it included one item originally associated with *Aligning Expectations* that emphasizes consideration of personal and professional differences that may impact mentor-mentee dynamics. Despite this inclusion, the core of this factor remains highly consistent with the original domain, justifying the retention of the label *Maintaining Effective Communication*.

#### Factor 2: Assessing understanding and fostering independence.

The second factor combines two originally distinct MCA domains: *Assessing Understanding* and *Fostering Independence*. It includes items that assess a mentor’s ability to accurately evaluate a mentee’s research capabilities, enhance understanding of research concepts, and support various aspects of research development such as motivation, confidence, and creativity. This combined factor shows how mentors help mentees grow both intellectually and emotionally during their research careers. We combine two existing factors from the original MCA and label this factor *Assessing Understanding and Fostering Independence* to reflect the dual emphasis on skill appraisal and empowerment.

#### Factor 3: Promoting professional development.

**Fig 2:**
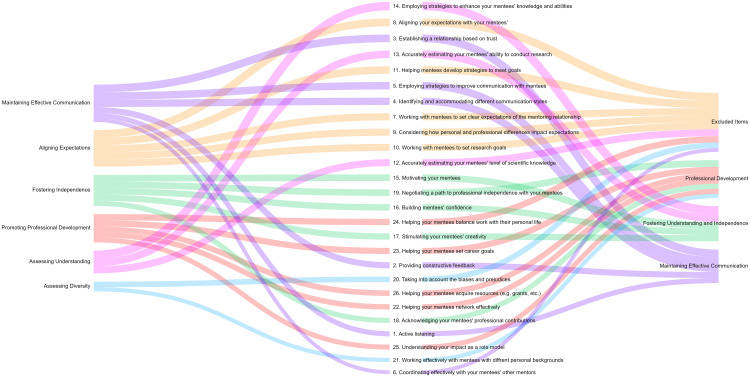
Sankey Diagram of MCA items (Fleming to proposed structure).

The third factor includes items from the original *Promoting Professional Development* domain along with others that emphasize professional identity formation, career planning, networking, and work-life integration. It also includes items that capture mentors’ attention to diversity and equity in career advancement contexts. As these elements reflect a broad and inclusive conception of mentoring for career progression, we retained the label *Promoting Professional Development*. Fig 2 provides a visual representation of the items construct structure as proposed in the original Fleming scale [19] to the new proposed structure.

#### Omitted original factors.

Notably, two of the six original MCA factors, *Aligning Expectations* and *Assessing Understanding,* are not discrete factors in the final 18-item model. Instead, the items associated with these domains are integrated within the remaining factors. For example, items originally tied to aligning expectations are now embedded within communication and diversity domains, and items assessing understanding have merged with those fostering independence. See Fig 2 for details about these omitted items. This also suggests that setting expectations or assessing knowledge may not be distinct mentoring competencies but rather are inherently part of the broader mentoring relationship.

## Discussion

This study revalidated the Mentoring Competency Assessment (MCA) using Exploratory Graph Analysis (EGA) with a diverse sample of mentors from a Hispanic-Serving Institution (HSI). Our analysis revealed a more parsimonious and conceptually coherent three-factor, 18-item model, offering a departure from the original six-factor, 26-item structure proposed by [19]. The three refined factors, *Maintaining Effective Communication*, *Assessing Understanding and Fostering Independence*, and *Promoting Professional Development*, align with core mentoring domains and offer a model that is more reliable and structurally sound than the original MCA.

Addressing Research Question 2, the superiority of the data-driven 3-factor models (Leiden and Louvain) over the original Fleming (6-factor, 26-item) and revised Hyun (6-factor, 21-item) models in this sample is a critical finding. Specifically, the original models demonstrated poor fit indices (e.g., Robust CFI **≤** 0.83, Robust RMSEA **≥** 0.12), suggesting their hypothesized factor structures do not adequately capture the underlying dimensionality of the MCA items for mentors at this HSI. This contrasts sharply with the preferred Leiden 3-factor model, which exceeded the acceptable CFI threshold (Robust CFI = 0.91). This explicit departure from the established structures suggests that mentoring competencies may be organized more parsimoniously, and perhaps more contextually, within institutions serving diverse populations, warranting a simpler structure than what was derived from the samples of the prior validations.

Two originally proposed domains, *Aligning Expectations* and *Assessing Understanding*, were not represented as distinct factors in our EGA-derived structure. Instead, the competencies typically associated with these factors were integrated within broader relational and developmental processes. This suggests that, although expectation setting and knowledge assessment are foundational mentoring skills, they may not operate independently in practice [5,17]. Ultimately, the EGA approach allowed for a more flexible and data-driven understanding of how these competencies co-occur in real-world mentoring relationships.

### Implications for mentorship evaluation and training

The results of this study offer a significant contribution to the literature by demonstrating the limitations of previous, more complex factor structures [5, 19] when applied to the unique context of an HSI, thereby addressing the core assessment gap outlined in the Introduction. Our development of a revised, more streamlined MCA (18-item, 3-factor model) critiques the generalizability of prior MCA validations and builds upon the literature by providing a *parsimonious, context-specific measure*. This abbreviated structure is empirically supported and offers a streamlined tool for assessing mentor development, enhancing its utility in institutional assessment and professional development contexts, particularly for programs that serve historically underrepresented students. For programs that serve historically underrepresented students, particularly those at MSIs, this abbreviated structure may offer a culturally relevant and less burdensome way to capture key mentoring competencies [31].

This abbreviated, parsimonious structure is particularly beneficial for institutional use at MSIs. At this HSI, which serves a student population with high rates of first-generation status, bilingualism, and low socioeconomic status, faculty mentors often manage significant service loads and time constraints. A 3-factor, 18-item instrument is demonstrably easier to administer and interpret compared to the original 26-item, 6-factor structure, promoting greater compliance and utility in a high-volume research environment. Furthermore, the resulting 3-factor structure is culturally relevant because it consolidates competence domains. For instance, the clustering of diversity-related items (e.g., ‘Considering how personal and professional differences may impact expectations’) into the Maintaining Effective Communication factor reflects that culturally responsive practice in this bicultural HSI context is not an add-on, but an essential component of the communication and relationship-building processes, as suggested by literature on culturally responsive mentoring.

Importantly, our structure also reinforces the view that culturally responsive mentoring, acknowledging mentees’ diverse backgrounds and building inclusive communication practices, for example, is not a separate domain, but rather embedded throughout mentoring relationships [11,31]. For our final model, items tied to diversity considerations clustered within the communication and professional development factors, suggesting that equity and inclusion are foundational to effective mentorship. The study confirmed that diversity skills are fundamental, not separate. When analyzed, the skill related to the item “Considering how personal and professional differences may impact expectations” clustered directly with the essential factor of Maintaining Effective Communication. In a similar finding, the item “Working effectively with mentees whose personal background is different from his/her own” grouped with the factor Promoting Professional Development. The CFA confirmed that equity and inclusion are embedded within core mentoring effectiveness, rather than being isolated skills. Furthermore, the first factor (Communication) and the third factor (Professional Development) are highly interconnected (strong positive correlation), meaning these skills often operate together in practice.

Based on the refined 3-factor model, we recommend that HSI-specific mentor training programs focus on three core areas, rather than the six originally proposed. First, training should explicitly integrate cultural humility into sessions on Maintaining Effective Communication, teaching mentors how to manage expectations and communicate constructively while explicitly considering mentees’ personal, professional, and cultural differences (as captured by the inclusion of MCA item 9). Second, training related to Assessing Understanding and Fostering Independence should be combined to emphasize the iterative cycle of assessing skill, building confidence, and then granting independence. Third, the Promoting Professional Development training should not be limited to career planning, but must explicitly include topics on how to be an effective role model and leverage one’s network to actively facilitate career progression and access to resources for mentees from marginalized backgrounds.

### Conclusion

This revalidation study successfully established a parsimonious, context-specific 3-factor structure for the Mentoring Competency Assessment (MCA-18), offering a psychometrically rigorous tool for evaluation and training within Hispanic-Serving Institutions. The empirical support for this revised structure, particularly the high model fit indices (Robust CFI = 0.91) and the successful consolidation of items across factors, directly justifies the conceptual claim that culturally responsive practice is an *embedded* and *essential* component of core mentoring competencies at this HSI. This finding suggests that cultural responsiveness is intrinsically linked to effective relational practice in this HSI context. The resulting model addresses the core assessment gap by providing a clearer, more efficient, and more relevant framework for mentor evaluation and training tailored to support diverse student populations. Nevertheless, the generalizability of these findings remains constrained by the single-institution sample, indicating a need for multi-site replication to confirm the stability of the 3-factor structure.

### Limitations

Several limitations should be acknowledged. Although the sample was drawn from a large, research intensive HSI and represents an understudied population, it is limited to a single institution. Generalizability to other HSIs, MSIs, or to predominantly White institutions should be tested in future research. Second, the MCA data were self-reported by mentors, which may introduce social desirability or recall bias. Including mentee assessments or independent observations would provide a more comprehensive picture [32]. Third, this reliance on self-report data also raises the possibility of Common Method Bias (CMB), a form of systematic error that threatens the validity of observed relationships. While CMB was not formally assessed, the high measurement reliability confirmed in our CFA reduces the concern about this specific bias [33]. Fourth, although EGA is useful for identifying community structures and is gaining support in psychometrics [28], it should be complemented with theory-based validation, including longitudinal and predictive analyses, to ensure the revised structure works as intended over time and with interventions. Finally, we were unable to analyze the impact of key mentor characteristics such as race/ethnicity, gender identity, or academic discipline/career stage on the resulting factor structure. This specific data gap stems from a lack of self-report for these demographic variables, and the inability to run analyses due to the significantly reduced sample sizes available for any subsequent stratified analysis.

### Future directions

Further research is required to fully validate the stability and predictive utility of the streamlined MCA-18. To establish generalizability, the 18-item model should be tested across a range of academic disciplines and institutional contexts, including community colleges and predominantly White institutions, to assess whether this parsimonious structure remains robust outside of the initial HSI context. Furthermore, to enhance the scale’s validity, future studies should move beyond mentor self-report by integrating mentee perspectives (multi-source data) and employing longitudinal designs. Analyses that examine mentor-mentee dyads, in particular, could offer valuable insight into mentor-mentee alignment and help evaluate the measure’s predictive validity for critical mentee outcomes like science identity, research self-efficacy, and persistence in STEM.

Beyond psychometric validation, future scale adaptations might consider incorporating culturally specific mentoring practices relevant to MSIs, such as *familismo*, bilingual communication, intergenerational guidance, or community-connectedness. Integrating these culturally resonant constructs would ensure the MCA’s full cultural validity and responsiveness, providing a tool that better reflects the mentoring values and norms within diverse student populations and strengthens its utility in equity-focused research training

## Supporting information

S1 Table Mentor Demographics. (DOCX)

S2 Table Standardized CFA Loadings. (DOCX)

S1 Fig Scree Plot of Modified Parallel Analysis. (PNG)

S2 Fig Network Plot of GLASSO EGA with Louvain algorithm (19-item). (PNG)

S3 Fig Item Stability Plot of GLASSO EGA with Leiden algorithm (18-item). (PNG)

S4 Fig Item Stability Plot of GLASSO EGA with Louvain algorithm (19-item). (PNG)

S1 File De-identified Data. (XLSX)
