# Supplementary material for: Assessing undergraduate mentoring competency in a research-intensive Hispanic serving institution: A revalidation
Source: PLoS One. 2026 Jun 25;21(6):e0350417. doi: 10.1371/journal.pone.0350417 (PMC13298900; doi:10.1371/journal.pone.0350417)
Supplement: S1 Table — (DOCX) [file pone.0350417.s001.docx]

## SI Table . Mentor Demographics

|  | *n* | *%* |
| --- | --- | --- |
| Academic rank |  |  |
| Assistant Professor | 85 | 24.1% |
| Associate Professor | 114 | 32.4% |
| Director | 1 | 0.3% |
| Graduate Student | 13 | 3.7% |
| Lecturer | 1 | 0.3% |
| PhD Student | 14 | 4.0% |
| Post-doc | 10 | 2.8% |
| Professor | 106 | 30.1% |
| Research Assistant Professor | 1 | 0.3% |
| Research Associate Professor | 2 | 0.6% |
| Researcher | 3 | 0.9% |
| Staff | 2 | 0.6% |
| Gender |  |  |
| Male | 43 | 53.1% |
| Female | 37 | 45.7% |
| Other | 1 | 1.2% |
| Participation Year |  |  |
| 2017 | 42 | 11.7% |
| 2018 | 74 | 20.6% |
| 2019 | 83 | 23.1% |
| 2020 | 24 | 6.7% |
| 2021 | 60 | 16.7% |
| 2022 | 41 | 11.4% |
| 2023 | 25 | 6.9% |
| 2024 | 11 | 3.1% |

N = 363
