# Supplementary material for: Assessing undergraduate mentoring competency in a research-intensive Hispanic serving institution: A revalidation
Source: PLoS One. 2026 Jun 25;21(6):e0350417. doi: 10.1371/journal.pone.0350417 (PMC13298900; doi:10.1371/journal.pone.0350417)
Supplement: S2 Table — (DOCX) [file pone.0350417.s002.docx]

## S2 Table. Standardized CFA Loadings

|  | **Fleming (6f, 26i)** | | | | | | **Hyun (6f, 21i)** | | | | | | **Louvain (3f, 19i)** | | | **Leiden (3f, 18i)** | | |
| --- | --- | --- | --- | --- | --- | --- | --- | --- | --- | --- | --- | --- | --- | --- | --- | --- | --- | --- |
|  | *1* | *2* | *3* | *4* | *5* | *6* | *1* | *2* | *3* | *4* | *5* | *6* | *1* | *2* | *3* | *1* | *2* | *3* |
| *MCA item* |  |  |  |  |  |  |  |  |  |  |  |  |  |  |  |  |  |  |
| 1. Active listening | 0.773 |  |  |  |  |  | 0.780 |  |  |  |  |  | 0.763 |  |  | 0.765 |  |  |
| 2. Constructive feedback | 0.786 |  |  |  |  |  | 0.794 |  |  |  |  |  | 0.763 |  |  | 0.767 |  |  |
| 3. Trust-based relationships | 0.869 |  |  |  |  |  | 0.866 |  |  |  |  |  | 0.872 |  |  | 0.872 |  |  |
| 4. Adapting communication | 0.886 |  |  |  |  |  | 0.875 |  |  |  |  |  | 0.895 |  |  | 0.892 |  |  |
| 5. Improving communication | 0.931 |  |  |  |  |  |  |  |  |  |  |  | 0.930 |  |  | 0.932 |  |  |
| 6. Co-mentor coordination | 0.509 |  |  |  |  |  |  |  |  |  |  |  |  |  |  |  |  |  |
| 7. Setting relationship expectations | | 0.824 |  |  |  |  |  | 0.799 |  |  |  |  |  |  |  |  |  |  |
| 8. Aligning expectations |  | 0.905 |  |  |  |  |  | 0.877 |  |  |  |  |  |  |  |  |  |  |
| 9. Considering differences |  | 0.845 |  |  |  |  |  |  |  |  | 0.862 |  | 0.838 |  |  | 0.834 |  |  |
| 10. Setting research goals |  | 0.889 |  |  |  |  |  |  | 0.890 |  |  |  |  |  |  |  |  |  |
| 11. Developing goal strategies |  | 0.896 |  |  |  |  |  |  | 0.895 |  |  |  |  |  |  |  |  |  |
| 12. Assessing scientific knowledge | |  | 0.885 |  |  |  |  |  | 0.842 |  |  |  |  |  |  |  |  |  |
| 13. Assessing research ability |  |  | 0.921 |  |  |  |  | 0.849 |  |  |  |  |  | 0.796 |  |  | 0.796 |  |
| 14. Enhancing knowledge & abilities | |  | 0.957 |  |  |  |  | 0.824 |  |  |  |  |  | 0.865 |  |  | 0.865 |  |
| 15. Motivating mentees |  |  |  | 0.826 |  |  |  |  |  |  |  |  |  | 0.850 |  |  | 0.848 |  |
| 16. Building confidence |  |  |  | 0.849 |  |  |  |  |  |  |  |  |  | 0.869 |  |  | 0.871 |  |
| 17. Stimulating creativity |  |  |  | 0.770 |  |  |  |  |  | 0.735 |  |  |  | 0.783 |  |  | 0.785 |  |
| 18. Acknowledging contributions | |  |  | 0.742 |  |  |  |  |  | 0.719 |  |  |  |  | 0.763 |  |  | 0.764 |
| 19. Negotiating independence |  |  |  | 0.744 |  |  |  |  |  | 0.726 |  |  |  |  | 0.784 |  |  | 0.786 |
| 20. Considering biases & prejudices | |  |  |  | 0.736 |  |  |  |  |  | 0.725 |  |  |  | 0.740 |  |  |  |
| 21. Working with diverse backgrounds | | |  |  | 0.735 |  |  |  |  |  | 0.724 |  |  |  | 0.732 |  |  | 0.734 |
| 22. Facilitating networking |  |  |  |  |  | 0.737 |  |  |  |  |  | 0.753 |  |  | 0.722 |  |  | 0.725 |
| 23. Setting career goals |  |  |  |  |  | 0.765 |  |  |  |  |  | 0.787 |  |  | 0.747 |  |  | 0.752 |
| 24. Balancing work/life |  |  |  |  |  | 0.733 |  |  |  |  |  | 0.751 |  |  |  |  |  |  |
| 25. Understanding role model impact | |  |  |  |  | 0.784 |  |  |  |  |  |  |  |  | 0.758 |  |  | 0.763 |
| 26. Acquiring resources |  |  |  |  |  | 0.745 |  |  |  |  |  | 0.762 |  |  | 0.731 |  |  | 0.740 |
| *Inter-Factor Correlations* |  |  |  |  |  |  |  |  |  |  |  |  |  |  |  |  |  |  |
| F1 |  | 0.928 | 0.796 | 0.896 | 0.865 | 0.811 |  | 0.936 | 0.885 | 0.935 | 0.913 | 0.797 |  | 0.890 | 0.845 |  | 0.890 | 0.832 |
| F2 |  |  | 0.812 | 0.898 | 0.876 | 0.861 |  |  | 1.058 | 0.964 | 0.911 | 0.838 |  |  | 0.878 |  |  | 0.876 |
| F3 |  |  |  | 0.864 | 0.747 | 0.727 |  |  |  | 0.923 | 0.837 | 0.798 |  |  |  |  |  |  |
| F4 |  |  |  |  | 0.950 | 0.906 |  |  |  |  | 1.006 | 0.935 |  |  |  |  |  |  |
| F5 |  |  |  |  |  | 0.941 |  |  |  |  |  | 0.882 |  |  |  |  |  |  |
