# Supplementary figures and images for: Assessing undergraduate mentoring competency in a research-intensive Hispanic serving institution: A revalidation
Source: PLoS One. 2026 Jun 25;21(6):e0350417. doi: 10.1371/journal.pone.0350417 (PMC13298900; doi:10.1371/journal.pone.0350417)

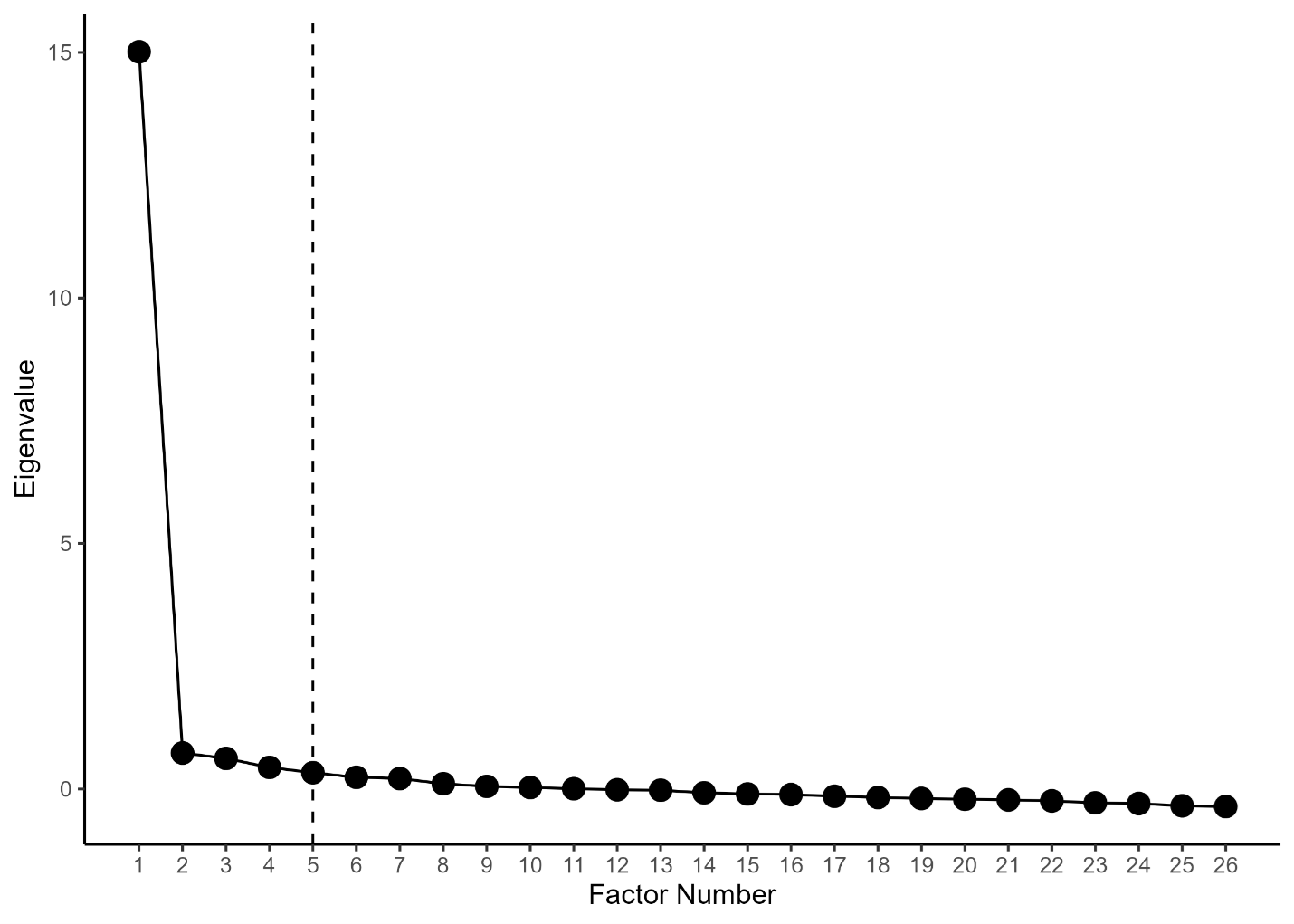

Supplement: S1 Fig — (PNG) [file pone.0350417.s003.png]

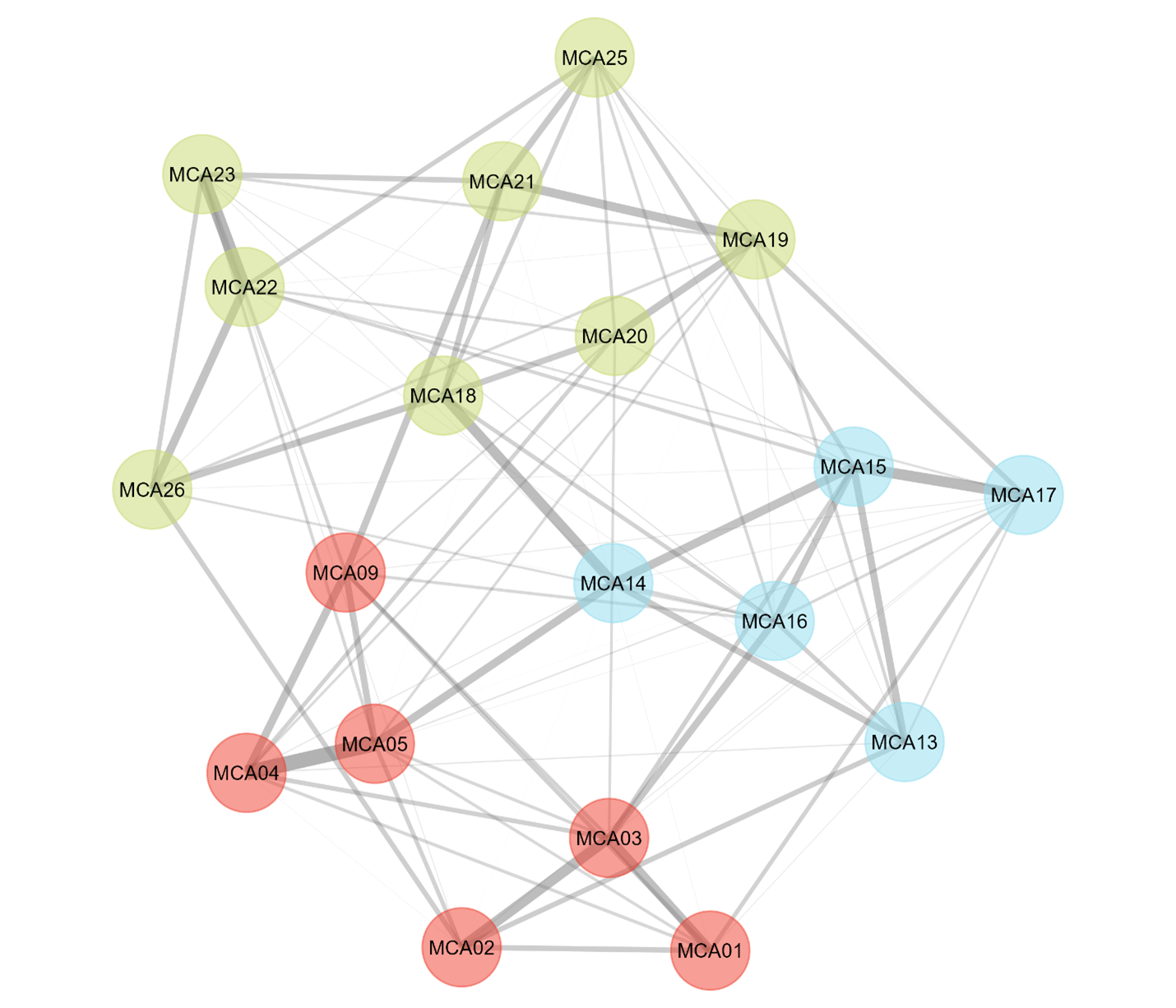

Supplement: S2 Fig — (PNG) [file pone.0350417.s004.png]

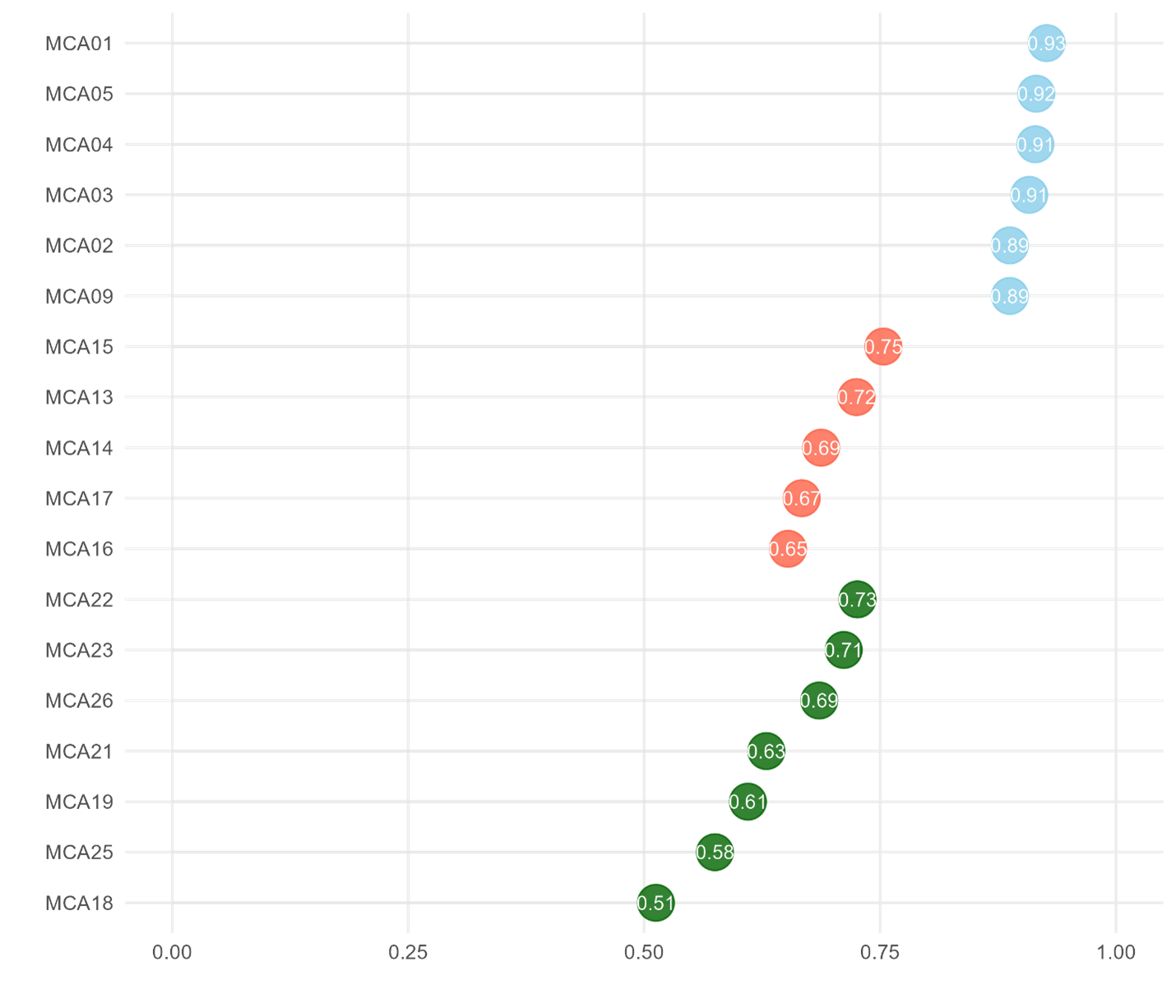

Supplement: S3 Fig — (PNG) [file pone.0350417.s005.png]

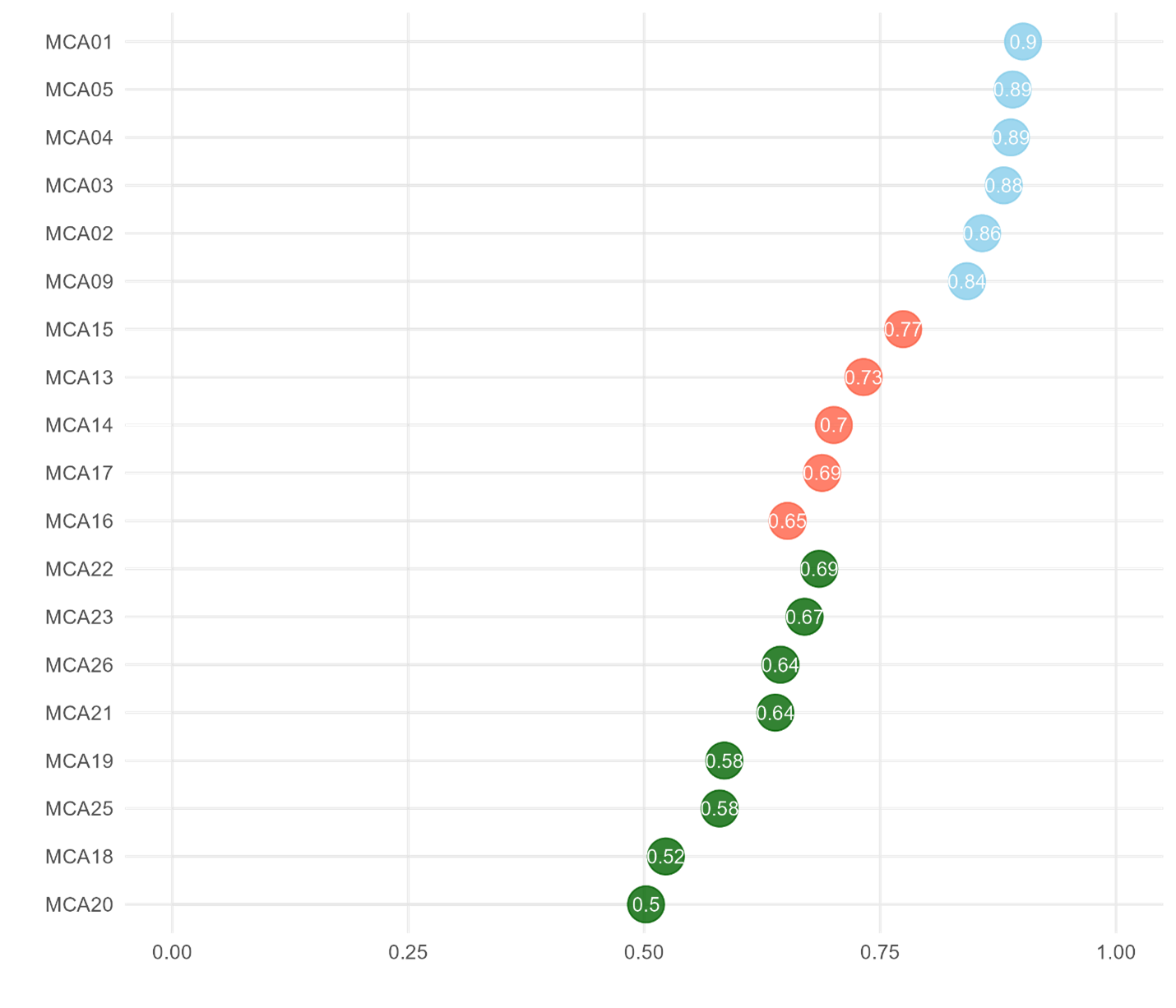

Supplement: S4 Fig — (PNG) [file pone.0350417.s006.png]
