# Supplementary material for: Assessing undergraduate mentoring competency in a research-intensive Hispanic serving institution: A revalidation
Source: PLoS One. 2026 Jun 25;21(6):e0350417. doi: 10.1371/journal.pone.0350417 (PMC13298900; doi:10.1371/journal.pone.0350417)
Supplement: Attachment — Submitted filename: Review comments.doc [file pone.0350417.s008.doc]

This study revalidated the MCA tool by applying EGA technology and proposed a more concise three-factor structure. This research offers a new perspective for the improvement of mentorship competency assessment tools, especially in institutions serving underrepresented minority students. However, the limitations of the study should not be overlooked, particularly in terms of sample representativeness and data collection methods. It is recommended that the authors consider the above comments in future revisions, further refine the research design and analysis methods, and enhance the reliability and generalizability of the research findings， and specific suggestions are as follows:

1. Although the study sample was drawn from a representative HSI, the sample was limited to a single institution, which may restrict the generalizability of the findings.

2. The study mentions sample diversity mainly in terms of race and ethnicity but does not elaborate on other factors that may affect mentoring competencies, such as mentors' disciplinary backgrounds and career stages. It is suggested that future research further explore the impact of these variables on the mentorship competency assessment tool.

3. The study utilized self-reported data from mentors, which may introduce social desirability or recall biases. It is recommended that future research consider incorporating assessments from mentees or independent observations to provide a more comprehensive evaluation perspective.

4. The results indicate that the original six-factor structure is not applicable to the new sample, instead forming a three-factor structure. This finding is significant for improving the mentorship competency assessment tool, but further validation is needed to confirm the stability of this structure across different disciplines and institutional contexts.

5. While the study proposes directions for future research, it is suggested to more specifically indicate how interdisciplinary and inter-institutional research can further validate and refine the MCA tool. Additionally, it is recommended to explore how to incorporate mentees' perspectives into the mentorship competency assessment and how to evaluate the predictive validity of the MCA tool through longitudinal studies.
