# Supplementary material for: Assessing undergraduate mentoring competency in a research-intensive Hispanic serving institution: A revalidation
Source: PLoS One. 2026 Jun 25;21(6):e0350417. doi: 10.1371/journal.pone.0350417 (PMC13298900; doi:10.1371/journal.pone.0350417)
Supplement: Attachment — Submitted filename: Reviewer Comments final.docx [file pone.0350417.s009.docx]

| **No** | **Review 1 Comments** | **Response** |
| --- | --- | --- |
| 1 | Thank you for the opportunity to review this paper, which picks up on a critical gap in understanding of the effectiveness of mentor effectiveness in diverse settings, specifically Hispanic serving institutions. This is an important area of growth, and the manuscript makes a valuable contribution to the field through the revalidation of the Mentoring Competency Assessment tool using EGA. I enjoyed reading this manuscript, and overall, it is well-written and accessible. However, I believe there are a few issues that require attention before the study can be published. I hope the notes here will serve to strengthen the paper. | Thank you! We appreciate your thoughtful reading and feedback. |
| 2 | The study rationale was properly placed in the context of previous literature, but there were quite a few instances of repetition in the introduction and literature review sections (e.g. in reference to Fleming et al.). It seemed a little odd to jump back to the literature after the rationale had been made. I wonder if the introduction and literature review sections could be merged to enhance readability? Note that the submission guidelines suggest introduction, then method, etc. | Thank you, that is a very good suggestion. The introduction and literature review have now been merged to a single introduction piece that includes the literature review as part of it, ending with the research questions and moving on to the method section. |
| 3 | Additionally, the only demographics reported for participants is year of participation, gender, and academic rank. I understand that the student population of the institution is 84.23% Hispanic, but this does not tell us about participants’ minoritized/ marginalized identities, just that they may mentor a higher than average proportion of Hispanic students. It is important to acknowledge how mentors’ own identities may have impacted on their self-reports, given the arguments/ rationale geared toward the cultural relevance of mentoring for minoritized and marginalised students. I don’t think this takes away from the importance of the study, but does need acknowledging. | Thank you, this is a good point. We assessed the ethnicity of the mentors and found that 58.5% of values were missing, but of those non-missing, 29.5% were Hispanic. This is now included in the paper. |
| 4 | I would expect to see a more developed discussion section. The manuscript jumps straight to the conclusion, and seems to be mostly a repetition of the results. Lacking is a discussion of what the results suggest about cultural diversity and relevance. Around line 554, the authors claim that the “abbreviated structure may offer a culturally relevant and less burdensome way to capture key mentoring competencies”; it would be good to see a discussion of how, i.e. in what ways is the new structure more culturally relevant? How will this help support marginalised and minoritised students in practice, specifically? | Thank you for this critical feedback. We expanded the discussion section to address how the refined three-factor structure (Research Skill Development, Identity and Belonging, and Career Alignment) reflect unique needs at an HSI. Specifically, in regard to ‘Identity and Belonging,’ we find that the items for ‘Addressing Diversity’ do not function as a standalone factor, but instead aligns with prior research that indicates that, for Latino and other minoritized populations, culture and diversity are not separate constructs, but rather central to their identity. We also added a discussion on how the 18-item reduces the burden for faculty at minority serving institutions (MSIs). |
| 5 | I recommend the implications section as a whole is in need of further development. Around line 282 of the manuscript, the authors argue “Successful interventions in this community are likely to benefit the broader academic community, as they address the needs of both minoritized (e.g., racial/ethnic minorities) and marginalized (e.g., low socioeconomic status) populations. Therefore, interventions that prove effective in this environment can be expected to work well in other settings” – how can the results be used to help support students from diverse communities? I understand the study was a revalidation study, but a short section highlighting some recommendations for mentors could increase the impact and utility of the study. | Thank you for the suggestion to increase the utility of the study for mentors. We have added recommendations with implications for mentoring. Specifically, we translate our three-factor structure of the mentoring competency scale into specific practices, such as prioritizing ‘Identity and Belonging’ through cultural humility and explicitly expanding discussion of professional development beyond career planning, but situating it in the context of socio-economic challenges faced by many students at HSIs and helping them understand topics on how to be effective role models and leveraging their networks. |
| 6 | The conclusion makes some claims that I’m not sure are fully supported by the results, as they are currently presented, e.g. “Importantly, our structure also reinforces the view that culturally responsive mentoring, acknowledging mentees' diverse backgrounds and building inclusive communication practices, for example, is not a separate domain, but rather embedded throughout mentoring relationships” (lines 556-8), and “For our final model, items tied to diversity considerations clustered within the communication and professional development factors, suggesting that equity and inclusion are foundational to effective mentorship” (lines 599-61). More developed discussion of the results is needed here to link these claims to the results of the GFA. | The claims are now supported by demonstrating the integration of diversity-related items into the other core factors, and a discussion of the results to link the clustering of diversity items to the conclusion that equity and inclusion are foundational to effective mentorship |
| 7 | A minor point that the journal requires referencing in Vancouver style | Thank you for this suggestion. References have now been converted to Vancouver style. |
|  | **Review 2 Comments** |  |
| 8 | 1. The writing is verbose and repetitive, necessitating content reduction. For instance, the abstract should only introduce the research objectives, methods, results, and conclusions, with a focus on highlighting the author's own work. The work of other scholars can be briefly introduced as research background, rather than being extensively elaborated upon. There is no need for lengthy introductions of other scholars' work. Additionally, the extensive introduction of PCA and ICA in the "Statistical Approach" section is unnecessary. It is recommended to streamline this section and merge it with the "Comparing PCA and FA to EGA" section. | Thank you for this feedback. We agree and have streamlined the manuscript for clarity. The abstract has been revised to focus strictly on our objectives, the EGA methodology, and the key finding of a three-factor structure. We significantly reduced the theoretical background of PCA and ICA, only keeping the “Comparing PCA and FA to EGA” sub-section. This allows the reader to focus on the current study's application of network-based methods rather than a long discussion of historical statistical techniques. |
| 9 | 2. The overall logic of the article is unclear, and the structure is somewhat disorganized, requiring careful reorganization. For example, the paper emphasizes the application of the original Mentoring Competency Assessment in clinical and biomedical research contexts, but the relationship between this and the current study is unclear. This paper merely extends this MCA to Hispanic contexts for re-evaluation, without sufficiently analyzing the expansion of MCA's application across disciplines. Furthermore, the literature review specifically mentions that "Hyun and colleagues (2022) conducted a large-scale revalidation of the MCA using data from 1,626 mentors across a wider range of research institutions and disciplines," indicating the importance of Hyun's work. However, since this paper does not build upon Hyun's work nor compare its findings with Hyun's, it is unclear why the author devotes significant space to introducing Hyun's work. Additionally, the author does not analyze the reasons for the differences between the re-validated MCA and the original dimensions. In the methodology section, the author states that they used a 7-point Likert-type scale for the MCA, but it remains unclear whether Fleming et al.'s MCA also employed a 7-point scale. | We have reorganized the literature review to better bridge the gap between previous validation studies and our own. We included Fleming (2013) and Hyun (2022) to establish the current 'Gold Standard' (6-factor model) and to highlight that even large-scale revalidations (Hyun, 2022) have relied on traditional CFA/SEM. Our study builds upon their work by testing if those same six factors hold true in a specific cultural context (HSI) using a novel methodology (EGA). We have added a comparison in the Discussion section detailing why our 3-factor structure emerged in contrast to their 6-factor structure, specifically noting that in our sample, 'Diversity' and 'Communication' items collapsed into 'Identity and Belonging.' Finally, we clarified that the 7-point Likert scale used is consistent with the original MCA developed by Fleming et al. |
| 10 | 3. The methodology of this paper requires further clarification. For instance, it is uncertain whether the language of the questionnaire used in the Hispanic Serving Institutions matches the language of the research participants. I apologize for my lack of familiarity with the situation in the United States as a foreigner, and I would appreciate it if the author could clarify this point. Additionally, with only 323 valid participants in this study, the sample size is relatively small. The author should either increase the number of questions to expand the sample size or provide an explanation and declare this limitation in the research limitations section. | We have clarified in the 'Participants' section that the survey was administered in English, which is the primary language of instruction and professional research at this HSI. Regarding sample size, while N=323 is smaller than some national studies, it exceeds the requirements for stable Exploratory Graph Analysis (EGA). To address the reviewer’s concern, we performed a post-hoc Monte Carlo power analysis which demonstrated that our sample size provided 100% power to detect the identified latent structure. We have also added this as a limitation regarding generalizability to non-English speaking or non-HSI contexts |
| 11 | 4. Although the paper lists a substantial amount of work by other scholars in the field of MCA, the author fails to compare and analyze their own work with that of others, nor do they explain how their work critiques or builds upon the work of others. Consequently, it is difficult to assess the creativity of this study. | The creativity of this study lies in the intersection of cultural context and advanced psychometrics. By applying EGA we move beyond the traditional factor analysis. Our study critiques the 6-factor model by showing that for mentors at HSIs, competencies like 'Addressing Diversity' are not siloed skills but are fundamentally integrated into 'Identity and Belonging.' This provides a more culturally nuanced and 'less burdensome' 18-item tool for future research in diverse settings. |
|  | **Review 3 Comments** |  |
| 12 | This study revalidated the MCA tool by applying EGA technology and proposed a more concise three-factor structure. This research offers a new perspective for the improvement of mentorship competency assessment tools, especially in institutions serving underrepresented minority students. However, the limitations of the study should not be overlooked, particularly in terms of sample representativeness and data collection methods. It is recommended that the authors consider the above comments in future revisions, further refine the research design and analysis methods, and enhance the reliability and generalizability of the research findings. | We thank the reviewer for their positive assessment of the value of our work at Hispanic-Serving Institutions. We have taken the concerns regarding sample representativeness and data collection methods seriously. Specifically, we have:   1. Expanded the Limitations section to address the single-institution nature of the study and the use of self-reported data. 2. Added a Future Research section that outlines a roadmap for multi-institutional and longitudinal validation. 3. Provided a Post-hoc Power Analysis to support the reliability of our findings despite the smaller sample size relative to national studies |
| 13 | 1. Although the study sample was drawn from a representative HSI, the sample was limited to a single institution, which may restrict the generalizability of the findings. | We agree that the single-institution nature of this study is a limitation. While our institution is highly representative of HSIs in the Southwest, we have updated the Limitations section to explicitly state that the three-factor structure should be viewed as a 'context-specific model.' We also indicate in future directions that future multi-site studies to investigate if this structure holds across other HSIs and/or MSIs with different regional or demographic profiles. |
| 14 | 2. The study mentions sample diversity mainly in terms of race and ethnicity but does not elaborate on other factors that may affect mentoring competencies, such as mentors' disciplinary backgrounds and career stages. It is suggested that future research further explore the impact of these variables on the mentorship competency assessment tool. | This is an excellent point. While our primary focus was the cultural context of an HSI, factors such as disciplinary areas and career stage likely influence mentoring priorities. We have added this to our 'Future Directions' section, suggesting that future EGA models include these variables as covariates to see if the network structure shifts based on professional experience or discipline |
| 15 | 3. The study utilized self-reported data from mentors, which may introduce social desirability or recall biases. It is recommended that future research consider incorporating assessments from mentees or independent observations to provide a more comprehensive evaluation perspective. | We acknowledge that self-report data is subject to social desirability and recall bias. We have noted this in the Limitations section. We have also added a recommendation for pairing with student assessments, as well as conducting studies using a longitudinal framework. |
| 16 | 4. The results indicate that the original six-factor structure is not applicable to the new sample, instead forming a three-factor structure. This finding is significant for improving the mentorship competency assessment tool, but further validation is needed to confirm the stability of this structure across different disciplines and institutional contexts. | We agree. While our study successfully identified a new three-factor structure for this HSI population, we explicitly state in our limitations and future directions that this should be viewed as a 'context-specific' model, and that further validation is needed to see if this same structure holds true at other types of institutions or longitudinally. |
| 17 | 5. While the study proposes directions for future research, it is suggested to more specifically indicate how interdisciplinary and inter-institutional research can further validate and refine the MCA tool. Additionally, it is recommended to explore how to incorporate mentees' perspectives into the mentorship competency assessment and how to evaluate the predictive validity of the MCA tool through longitudinal studies. | Thank you for these suggestions. Our Future Research section now highlights these exact paths. We specify that the next logical steps for this work include:   1. Multi-site/Inter-institutional studies to see if the 18-item version works across different campuses. 2. Mentee perspectives, as our current study focused on mentor self-reports. 3. Longitudinal research to see if these 18-item scores actually predict long-term student success and persistence in STEM |
|  | **Reviewer 4 Comments** |  |
| 18 | The manuscript rationally explained the significance of the research and the needs of the literature. Current literature was consulted. The tool this revalidation research will provide to the field will contribute to mentoring research, particularly in culturally sensitive contexts. | We thank the Reviewer for their positive assessment of our manuscript and for recognizing the contribution this revalidated tool makes to mentoring research, particularly within culturally sensitive contexts like Hispanic-Serving Institutions (HSIs). |
|  | **Reviewer 5 Comments** |  |
| 19 | At first glance, this manuscript appears more like a technical report or project summary rather than a standard empirical research article suitable for an international peer-reviewed journal. The structure deviates significantly from the conventional academic format expected by journals such as Educational Researcher, or Studies in Higher Education. | We appreciate the feedback regarding the overall tone and structure of the manuscript. We have revised the document to move away from a 'project summary' format and have reorganized it into a standard empirical research structure (Introduction, Methods, Results, and Discussion). We have also refined the language throughout to ensure it meets the academic conventions and scholarly rigor expected by international peer-reviewed journals. |
| 20 | Specifically, the manuscript lacks clear and consistent sectioning (Abstract, Introduction, Research Questions, Methods, Results, Discussion, Conclusion) and does not follow a well-defined IMRaD (Introduction–Methods–Results–Discussion) structure. This organizational issue makes the paper difficult to follow and reduces its scholarly readability.  ****The document reads more like a project report rather than a cohesive research manuscript.  There is no properly formatted Abstract at the beginning. While an abstract is present in the submission form, it does not appear within the main text of the paper as per journal standards.  The Introduction section lacks clear subsections leading to defined research questions (RQs) or hypotheses.  The objectives are mentioned (pp. 13–14), but explicit research questions or hypotheses are missing. My Recommendation: Reorganize the manuscript according to international academic conventions:  Abstract – concise summary (250–300 words) including rationale, methods, results, and implications.  Introduction – establish the problem, literature gap, theoretical basis, and clearly state research questions (RQs) or hypotheses.  Methods – describe design, participants, instruments, data collection, and analysis procedures.  Results – present main findings with tables/figures.  Discussion – interpret findings, compare with literature, note limitations.  Conclusion and Implications – summarize contributions and suggest future research. | We appreciate this feedback regarding the organizational flow. We have restructured the manuscript to strictly follow the IMRaD (Introduction-Methods-Results-Discussion) convention as suggested. This includes clearly defined section headings and a cohesive narrative that transitions from the literature gap to our specific results. Additionally, we have corrected the formatting to include the Abstract within the main manuscript file. We have also followed the reviewer's recommendation to end the Introduction with a clear, numbered list of research questions. These questions focus on (1) the structural validity of the MCA in an HSI context, (2) the comparison between EGA and traditional methods, and (3) the implications of a more parsimonious factor structure. |
| 21 | ****The research gap is explained narratively but could be summarized more clearly in bullet or paragraph form that directly leads to research questions. so, Conclude the introduction with three or four numbered research questions. | We have revised the end of the Introduction to more clearly summarize the research gap. Following your suggestion, we have concluded this section with a numbered list of three specific research questions that lead directly into our study's objectives |
| 22 | Weaknesses:  No sampling strategy or power analysis is reported.  No pilot testing mentioned prior to analysis.  Common Method Bias (CMB) is not assessed, though self-report instruments are known to be prone to it.  Some sections (pp. 23–26) are overly technical, making them difficult for non-statistical readers.  Read and consult these studies , will help you rigor method and overall struture your manuscript and must cite them in your method section for Common Method Bias; <https://doi.org/10.1057/s41599-025-05584-3> , For Structure <https://doi.org/10.1186/s40359-025-02767-0> , <https://doi.org/10.1111/ssm.18381> | No pilot testing occurred; everything else addressed, including the power analysis using Monte Carlo simulation. |
