# Supplementary material for: Assessing undergraduate mentoring competency in a research-intensive Hispanic serving institution: A revalidation
Source: PLoS One. 2026 Jun 25;21(6):e0350417. doi: 10.1371/journal.pone.0350417 (PMC13298900; doi:10.1371/journal.pone.0350417)
Supplement: Attachment — Submitted filename: Mentoring at a HSI.docx [file pone.0350417.s010.docx]

No data in abstract

Do not use PWI----it is used a few times but often you spell it out and then abbreviate it again. Simply use the original wrding

Lit review is integrated into the first part—do not repeat it again

One of the most comprehensive tools, the MCA, was developed by given this sentence frame you need the authors name here[19]-----or rephrase this sentence is a better solution. It worked before the numbers but not now

Indent 5 spaces all paragraphs

This below is not adding to your argument:

Effective mentoring is crucial for the development of research competencies in early career researchers [19]. Mentors provide guidance, support, and knowledge to mentees. High-quality mentoring relationships are associated with better academic and career outcomes for mentees [20][21].

I suggest deleting the whole thing

Add citations for these : The Walktrap, Leiden, and Louvain algorithms.

Which was the sample size—they do not agree in the data analytic vs results sections

final analytical sample size of N = 323 OR The final analyzed sample consisted of N = 363 mentors

I don’t see a description of this: modified parallel analysis

Problem with this sentence:

A post-hoc power analysis conducted for this model using Monte Carlo simulation.
