# Supplementary material for: Assessing undergraduate mentoring competency in a research-intensive Hispanic serving institution: A revalidation
Source: PLoS One. 2026 Jun 25;21(6):e0350417. doi: 10.1371/journal.pone.0350417 (PMC13298900; doi:10.1371/journal.pone.0350417)
Supplement: Attachment — Submitted filename: Response to Reviewers.docx [file pone.0350417.s011.docx]

No data in abstract

Thank you, the citation about the final model CLI is now added.

Do not use PWI----it is used a few times but often you spell it out and then abbreviate it again. Simply use the original wrding

Thank you again, This is now corrected in the manuscript.

Lit review is integrated into the first part—do not repeat it again

Thank you for this-now fully integrated.

One of the most comprehensive tools, the MCA, was developed by given this sentence frame you need the authors name here[19]-----or rephrase this sentence is a better solution. It worked before the numbers but not now

This sentence is now corrected in the manuscript.

Indent 5 spaces all paragraphs

All paragraphs are now indented. Thank you.

This below is not adding to your argument:

Effective mentoring is crucial for the development of research competencies in early career researchers [19]. Mentors provide guidance, support, and knowledge to mentees. High-quality mentoring relationships are associated with better academic and career outcomes for mentees [20][21].

I suggest deleting the whole thing

Thank you, this is now deleted as are references 20 and 21 given they were only cited here.

Add citations for these : The Walktrap, Leiden, and Louvain algorithms.

Thank you-this is now cited.

Which was the sample size—they do not agree in the data analytic vs results sections

final analytical sample size of N = 323 OR The final analyzed sample consisted of N = 363 mentors

This is corrected. The 323 was the final sample size and 363 was the size before item-wise deletion. The table figures now corrected and this is clarified in the paper.

I don’t see a description of this: modified parallel analysis

Thank you- this is now corrected and a description is added to methods.

Problem with this sentence:

A post-hoc power analysis conducted for this model using Monte Carlo simulation.

Thank you- this is now corrected with an appropriate verb added to the sentence.
