# Supplementary material for: Assessing undergraduate mentoring competency in a research-intensive Hispanic serving institution: A revalidation
Source: PLoS One. 2026 Jun 25;21(6):e0350417. doi: 10.1371/journal.pone.0350417 (PMC13298900; doi:10.1371/journal.pone.0350417)
Supplement: Peer review history

# Peer review: Decision Letter 0

**DOI:** 10.1371/journal.pone.0350417.r001

**Authors:** Nadia Rehman

9 Oct 2025

PONE-D-25-32929

Assessing Undergraduate Mentoring Competency in a Research-Intensive Hispanic Serving Institution: A Revalidation

PLOS ONE

Dear Dr. Wagler,

Thank you for submitting your manuscript to PLOS ONE. After careful consideration, we feel that it has merit but does not fully meet PLOS ONE’s publication criteria as it currently stands. Therefore, we invite you to submit a revised version of the manuscript that addresses the points raised during the review process.

==============================

Dear Authors,

Your manuscript has been reviewed. Based on the reviewers’ evaluation, I recommend major revisions before further consideration. Reviewers **2, 3, and 5**  have provided comprehensive and detailed comments, please ensure that all points raised are carefully addressed in your revision.

Kind regards,

==============================

Please submit your revised manuscript by Nov 23 2025 11:59PM. If you will need more time than this to complete your revisions, please reply to this message or contact the journal office at plosone@plos.org. When you're ready to submit your revision, log on to https://www.editorialmanager.com/pone/ and select the 'Submissions Needing Revision' folder to locate your manuscript file.

Please include the following items when submitting your revised manuscript:

- A rebuttal letter that responds to each point raised by the academic editor and reviewer(s). You should upload this letter as a separate file labeled 'Response to Reviewers'.
- A marked-up copy of your manuscript that highlights changes made to the original version. You should upload this as a separate file labeled 'Revised Manuscript with Track Changes'.
- An unmarked version of your revised paper without tracked changes. You should upload this as a separate file labeled 'Manuscript'.

If you would like to make changes to your financial disclosure, please include your updated statement in your cover letter. Guidelines for resubmitting your figure files are available below the reviewer comments at the end of this letter.

If applicable, we recommend that you deposit your laboratory protocols in protocols.io to enhance the reproducibility of your results. Protocols.io assigns your protocol its own identifier (DOI) so that it can be cited independently in the future. For instructions see: https://journals.plos.org/plosone/s/submission-guidelines#loc-laboratory-protocols. Additionally, PLOS ONE offers an option for publishing peer-reviewed Lab Protocol articles, which describe protocols hosted on protocols.io. Read more information on sharing protocols at https://plos.org/protocols?utm_medium=editorial-email&utm_source=authorletters&utm_campaign=protocols.

We look forward to receiving your revised manuscript.

Kind regards,

Nadia Rehman, Ph.D.

Academic Editor

PLOS ONE

Journal Requirements:

When submitting your revision, we need you to address these additional requirements.

1. Please ensure that your manuscript meets PLOS ONE's style requirements, including those for file naming. The PLOS ONE style templates can be found at https://journals.plos.org/plosone/s/file?id=wjVg/PLOSOne_formatting_sample_main_body.pdf and https://journals.plos.org/plosone/s/file?id=ba62/PLOSOne_formatting_sample_title_authors_affiliations.pdf

2. Please note that PLOS ONE has specific guidelines on code sharing for submissions in which author-generated code underpins the findings in the manuscript. In these cases, we expect all author-generated code to be made available without restrictions upon publication of the work. Please review our guidelines at https://journals.plos.org/plosone/s/materials-and-software-sharing#loc-sharing-code and ensure that your code is shared in a way that follows best practice and facilitates reproducibility and reuse.

3. Please include your full ethics statement in the ‘Methods’ section of your manuscript file. In your statement, please include the full name of the IRB or ethics committee who approved or waived your study, as well as whether or not you obtained informed written or verbal consent. If consent was waived for your study, please include this information in your statement as well.

4. We note that you have indicated that there are restrictions to data sharing for this study. For studies involving human research participant data or other sensitive data, we encourage authors to share de-identified or anonymized data. However, when data cannot be publicly shared for ethical reasons, we allow authors to make their data sets available upon request. For information on unacceptable data access restrictions, please see http://journals.plos.org/plosone/s/data-availability#loc-unacceptable-data-access-restrictions.

Before we proceed with your manuscript, please address the following prompts:

a) If there are ethical or legal restrictions on sharing a de-identified data set, please explain them in detail (e.g., data contain potentially identifying or sensitive patient information, data are owned by a third-party organization, etc.) and who has imposed them (e.g., a Research Ethics Committee or Institutional Review Board, etc.). Please also provide contact information for a data access committee, ethics committee, or other institutional body to which data requests may be sent.

b) If there are no restrictions, please upload the minimal anonymized data set necessary to replicate your study findings to a stable, public repository and provide us with the relevant URLs, DOIs, or accession numbers. Please see http://www.bmj.com/content/340/bmj.c181.long for guidelines on how to de-identify and prepare clinical data for publication. For a list of recommended repositories, please see https://journals.plos.org/plosone/s/recommended-repositories. You also have the option of uploading the data as Supporting Information files, but we would recommend depositing data directly to a data repository if possible.

Please update your Data Availability statement in the submission form accordingly.

5. In the online submission form, you indicated that data cannot be shared publicly to protect confidentiality of research participants. Data are available from the corresponding author upon request.

All PLOS journals now require all data underlying the findings described in their manuscript to be freely available to other researchers, either 1. In a public repository, 2. Within the manuscript itself, or 3. Uploaded as supplementary information.

This policy applies to all data except where public deposition would breach compliance with the protocol approved by your research ethics board. If your data cannot be made publicly available for ethical or legal reasons (e.g., public availability would compromise patient privacy), please explain your reasons on resubmission and your exemption request will be escalated for approval.

6. We note that the grant information you provided in the ‘Funding Information’ and ‘Financial Disclosure’ sections do not match.

When you resubmit, please ensure that you provide the correct grant numbers for the awards you received for your study in the ‘Funding Information’ section.

7. Thank you for stating the following financial disclosure:

Research reported in this paper was supported by the National Institute of General Medical Sciences of the National Institutes of Health under linked Award Numbers RL5GM118969, TL4GM118971, and UL1GM118970. The content is solely the responsibility of the authors and does not necessarily represent the official views of the National Institutes of Health.

Please state what role the funders took in the study. If the funders had no role, please state: "The funders had no role in study design, data collection and analysis, decision to publish, or preparation of the manuscript."

If this statement is not correct you must amend it as needed.

Please include this amended Role of Funder statement in your cover letter; we will change the online submission form on your behalf.

8. Please amend your manuscript to include your abstract after the title page.

9. Please include a copy of Table 2 which you refer to in your text on page 23.

10. We notice that your supplementary [figures/tables] are included in the manuscript file. Please remove them and upload them with the file type 'Supporting Information'. Please ensure that each Supporting Information file has a legend listed in the manuscript after the references list.

11. If the reviewer comments include a recommendation to cite specific previously published works, please review and evaluate these publications to determine whether they are relevant and should be cited. There is no requirement to cite these works unless the editor has indicated otherwise.

[Note: HTML markup is below. Please do not edit.]

Reviewers' comments:

Reviewer's Responses to Questions

**Comments to the Author**

1. Is the manuscript technically sound, and do the data support the conclusions?

The manuscript must describe a technically sound piece of scientific research with data that supports the conclusions. Experiments must have been conducted rigorously, with appropriate controls, replication, and sample sizes. The conclusions must be drawn appropriately based on the data presented.

Reviewer #1: Partly

Reviewer #2: Yes

Reviewer #3: Yes

Reviewer #4: Yes

Reviewer #5: Partly

**********

2. Has the statistical analysis been performed appropriately and rigorously?

Reviewer #1: Yes

Reviewer #2: Yes

Reviewer #3: Yes

Reviewer #4: Yes

Reviewer #5: Yes

**********

3. Have the authors made all data underlying the findings in their manuscript fully available?

The PLOS Data policy requires authors to make all data underlying the findings described in their manuscript fully available without restriction, with rare exception (please refer to the Data Availability Statement in the manuscript PDF file). The data should be provided as part of the manuscript or its supporting information, or deposited to a public repository. For example, in addition to summary statistics, the data points behind means, medians and variance measures should be available. If there are restrictions on publicly sharing data—e.g. participant privacy or use of data from a third party—those must be specified.

Reviewer #1: No

Reviewer #2: Yes

Reviewer #3: Yes

Reviewer #4: No

Reviewer #5: No

**********

4. Is the manuscript presented in an intelligible fashion and written in standard English?

PLOS ONE does not copyedit accepted manuscripts, so the language in submitted articles must be clear, correct, and unambiguous. Any typographical or grammatical errors should be corrected at revision, so please note any specific errors here.

Reviewer #1: Yes

Reviewer #2: Yes

Reviewer #3: Yes

Reviewer #4: Yes

Reviewer #5: Yes

**********

5. Review Comments to the Author

Please use the space provided to explain your answers to the questions above. You may also include additional comments for the author, including concerns about dual publication, research ethics, or publication ethics. (Please upload your review as an attachment if it exceeds 20,000 characters)

Reviewer #1: PONE-D-25-32929

"Assessing Undergraduate Mentoring Competency in a Research-Intensive Hispanic Serving Institution: A Revalidation"

Thank you for the opportunity to review this paper, which picks up on a critical gap in understanding of the effectiveness of mentor effectiveness in diverse settings, specifically Hispanic serving institutions. This is an important area of growth, and the manuscript makes a valuable contribution to the field through the revalidation of the Mentoring Competency Assessment tool using EGA. I enjoyed reading this manuscript, and overall, it is well-written and accessible. However, I believe there are a few issues that require attention before the study can be published. I hope the notes here will serve to strengthen the paper.

The study rationale was properly placed in the context of previous literature, but there were quite a few instances of repetition in the introduction and literature review sections (e.g. in reference to Fleming et al.). It seemed a little odd to jump back to the literature after the rationale had been made. I wonder if the introduction and literature review sections could be merged to enhance readability? Note that the submission guidelines suggest introduction, then method, etc.

There is reference to “a more recent revalidation effort by Hyun and colleagues (2022)” that “sought to refine the MCA using a larger, more diverse sample of research mentors” around line 66. I wondered how the sample of that study compared to the present study, and whether it would be helpful to readers to have this information.

The study rationale seems to be grounded in the premise that mentoring plays critical role in the development of research skills, professional identity, and career trajectories of undergraduate students, particularly those from underrepresented backgrounds. However, the study relies on data from mentors. Although it is noted in the limitations section that the data were self-reported by mentors, the authors only highlight this may introduce social desirability or recall bias. I would expect there to be some discussion of the importance of gaining the perspectives of mentees, since their perspectives is likely to differ.

Additionally, the only demographics reported for participants is year of participation, gender, and academic rank. I understand that the student population of the institution is 84.23% Hispanic, but this does not tell us about participants’ minoritized/ marginalized identities, just that they may mentor a higher than average proportion of Hispanic students. It is important to acknowledge how mentors’ own identities may have impacted on their self-reports, given the arguments/ rationale geared toward the cultural relevance of mentoring for minoritized and marginalised students. I don’t think this takes away from the importance of the study, but does need acknowledging.

I would expect to see a more developed discussion section. The manuscript jumps straight to the conclusion, and seems to be mostly a repetition of the results. Lacking is a discussion of what the results suggest about cultural diversity and relevance. Around line 554, the authors claim that the “abbreviated structure may offer a culturally relevant and less burdensome way to capture key mentoring competencies”; it would be good to see a discussion of how, i.e. in what ways is the new structure more culturally relevant? How will this help support marginalised and minoritised students in practice, specifically?

I recommend the implications section as a whole is in need of further development. Around line 282 of the manuscript, the authors argue “Successful interventions in this community are likely to benefit the broader academic community, as they address the needs of both minoritized (e.g., racial/ethnic minorities) and marginalized (e.g., low socioeconomic status) populations. Therefore, interventions that prove effective in this environment can be expected to work well in other settings” – how can the results be used to help support students from diverse communities? I understand the study was a revalidation study, but a short section highlighting some recommendations for mentors could increase the impact and utility of the study.

The conclusion makes some claims that I’m not sure are fully supported by the results, as they are currently presented, e.g. “Importantly, our structure also reinforces the view that culturally responsive mentoring, acknowledging mentees' diverse backgrounds and building inclusive communication practices, for example, is not a separate domain, but rather embedded throughout mentoring relationships” (lines 556-8), and “For our final model, items tied to diversity considerations clustered within the communication and professional development factors, suggesting that equity and inclusion are foundational to effective mentorship” (lines 599-61). More developed discussion of the results is needed here to link these claims to the results of the GFA.

A minor point that the journal requires referencing in Vancouver style

Reviewer #2: Dear editors:

The author re-validated the Mentoring Competency Assessment (MCA) using the Exploratory Graph Analysis method within Hispanic Serving Institutions, which holds theoretical significance for studying the external validity of MCA and practical significance for understanding mentoring systems within Hispanic contexts. However, the article has the following issues that require revision:

1. The writing is verbose and repetitive, necessitating content reduction. For instance, the abstract should only introduce the research objectives, methods, results, and conclusions, with a focus on highlighting the author's own work. The work of other scholars can be briefly introduced as research background, rather than being extensively elaborated upon. There is no need for lengthy introductions of other scholars' work. Additionally, the extensive introduction of PCA and ICA in the "Statistical Approach" section is unnecessary. It is recommended to streamline this section and merge it with the "Comparing PCA and FA to EGA" section.

2. The overall logic of the article is unclear, and the structure is somewhat disorganized, requiring careful reorganization. For example, the paper emphasizes the application of the original Mentoring Competency Assessment in clinical and biomedical research contexts, but the relationship between this and the current study is unclear. This paper merely extends this MCA to Hispanic contexts for re-evaluation, without sufficiently analyzing the expansion of MCA's application across disciplines. Furthermore, the literature review specifically mentions that "Hyun and colleagues (2022) conducted a large-scale revalidation of the MCA using data from 1,626 mentors across a wider range of research institutions and disciplines," indicating the importance of Hyun's work. However, since this paper does not build upon Hyun's work nor compare its findings with Hyun's, it is unclear why the author devotes significant space to introducing Hyun's work. Additionally, the author does not analyze the reasons for the differences between the re-validated MCA and the original dimensions. In the methodology section, the author states that they used a 7-point Likert-type scale for the MCA, but it remains unclear whether Fleming et al.'s MCA also employed a 7-point scale.

3. The methodology of this paper requires further clarification. For instance, it is uncertain whether the language of the questionnaire used in the Hispanic Serving Institutions matches the language of the research participants. I apologize for my lack of familiarity with the situation in the United States as a foreigner, and I would appreciate it if the author could clarify this point. Additionally, with only 323 valid participants in this study, the sample size is relatively small. The author should either increase the number of questions to expand the sample size or provide an explanation and declare this limitation in the research limitations section.

4. Although the paper lists a substantial amount of work by other scholars in the field of MCA, the author fails to compare and analyze their own work with that of others, nor do they explain how their work critiques or builds upon the work of others. Consequently, it is difficult to assess the creativity of this study.

In summury, This paper holds certain significance, but it still requires careful revision by the author.

Reviewer #3: This study revalidated the MCA tool by applying EGA technology and proposed a more concise three-factor structure. This research offers a new perspective for the improvement of mentorship competency assessment tools, especially in institutions serving underrepresented minority students. However, the limitations of the study should not be overlooked, particularly in terms of sample representativeness and data collection methods. It is recommended that the authors consider the above comments in future revisions, further refine the research design and analysis methods, and enhance the reliability and generalizability of the research findings.

Reviewer #4: The manuscript rationally explained the significance of the research and the needs of the literature. Current literature was consulted. The tool this revalidation research will provide to the field will contribute to mentoring research, particularly in culturally sensitive contexts.

Reviewer #5: At first glance, this manuscript appears more like a technical report or project summary rather than a standard empirical research article suitable for an international peer-reviewed journal. The structure deviates significantly from the conventional academic format expected by journals such as Educational Researcher, or Studies in Higher Education.

Specifically, the manuscript lacks clear and consistent sectioning (Abstract, Introduction, Research Questions, Methods, Results, Discussion, Conclusion) and does not follow a well-defined IMRaD (Introduction–Methods–Results–Discussion) structure. This organizational issue makes the paper difficult to follow and reduces its scholarly readability.

****The document reads more like a project report rather than a cohesive research manuscript.

There is no properly formatted Abstract at the beginning. While an abstract is present in the submission form, it does not appear within the main text of the paper as per journal standards.

The Introduction section lacks clear subsections leading to defined research questions (RQs) or hypotheses.

The objectives are mentioned (pp. 13–14), but explicit research questions or hypotheses are missing.

My Recommendation:

Reorganize the manuscript according to international academic conventions:

Abstract – concise summary (250–300 words) including rationale, methods, results, and implications.

Introduction – establish the problem, literature gap, theoretical basis, and clearly state research questions (RQs) or hypotheses.

Methods – describe design, participants, instruments, data collection, and analysis procedures.

Results – present main findings with tables/figures.

Discussion – interpret findings, compare with literature, note limitations.

Conclusion and Implications – summarize contributions and suggest future research.

****The research gap is explained narratively but could be summarized more clearly in bullet or paragraph form that directly leads to research questions. so, Conclude the introduction with three or four numbered research questions.

****Methodology

Weaknesses:

No sampling strategy or power analysis is reported.

No pilot testing mentioned prior to analysis.

Common Method Bias (CMB) is not assessed, though self-report instruments are known to be prone to it.

Some sections (pp. 23–26) are overly technical, making them difficult for non-statistical readers.

Read and consult these studies , will help you rigor method and overall struture your manuscript and must cite them in your method section for Common Method Bias; https://doi.org/10.1057/s41599-025-05584-3 , For Structure https://doi.org/10.1186/s40359-025-02767-0 , https://doi.org/10.1111/ssm.18381

Good luck with your revisions!

**********

6. PLOS authors have the option to publish the peer review history of their article (what does this mean?). If published, this will include your full peer review and any attached files.

If you choose “no”, your identity will remain anonymous but your review may still be made public.

**Do you want your identity to be public for this peer review?**  For information about this choice, including consent withdrawal, please see our Privacy Policy.

Reviewer #1: **Yes:** Suzanne Hamilton

Reviewer #2: No

Reviewer #3: No

Reviewer #4: No

Reviewer #5: No

**********

[NOTE: If reviewer comments were submitted as an attachment file, they will be attached to this email and accessible via the submission site. Please log into your account, locate the manuscript record, and check for the action link "View Attachments". If this link does not appear, there are no attachment files.]

While revising your submission, please upload your figure files to the Preflight Analysis and Conversion Engine (PACE) digital diagnostic tool, https://pacev2.apexcovantage.com/. PACE helps ensure that figures meet PLOS requirements. To use PACE, you must first register as a user. Registration is free. Then, login and navigate to the UPLOAD tab, where you will find detailed instructions on how to use the tool. If you encounter any issues or have any questions when using PACE, please email PLOS at figures@plos.org. Please note that Supporting Information files do not need this step.

Attachment Submitted filename: Review comments.doc

# Author comment: Author response to Decision Letter 1

**DOI:** 10.1371/journal.pone.0350417.r002

31 Mar 2026

No Review 1 Comments Response

1 Thank you for the opportunity to review this paper, which picks up on a critical gap in understanding of the effectiveness of mentor effectiveness in diverse settings, specifically Hispanic serving institutions. This is an important area of growth, and the manuscript makes a valuable contribution to the field through the revalidation of the Mentoring Competency Assessment tool using EGA. I enjoyed reading this manuscript, and overall, it is well-written and accessible. However, I believe there are a few issues that require attention before the study can be published. I hope the notes here will serve to strengthen the paper. Thank you! We appreciate your thoughtful reading and feedback.

2 The study rationale was properly placed in the context of previous literature, but there were quite a few instances of repetition in the introduction and literature review sections (e.g. in reference to Fleming et al.). It seemed a little odd to jump back to the literature after the rationale had been made. I wonder if the introduction and literature review sections could be merged to enhance readability? Note that the submission guidelines suggest introduction, then method, etc. Thank you, that is a very good suggestion. The introduction and literature review have now been merged to a single introduction piece that includes the literature review as part of it, ending with the research questions and moving on to the method section.

3 Additionally, the only demographics reported for participants is year of participation, gender, and academic rank. I understand that the student population of the institution is 84.23% Hispanic, but this does not tell us about participants’ minoritized/ marginalized identities, just that they may mentor a higher than average proportion of Hispanic students. It is important to acknowledge how mentors’ own identities may have impacted on their self-reports, given the arguments/ rationale geared toward the cultural relevance of mentoring for minoritized and marginalised students. I don’t think this takes away from the importance of the study, but does need acknowledging. Thank you, this is a good point. We assessed the ethnicity of the mentors and found that 58.5% of values were missing, but of those non-missing, 29.5% were Hispanic. This is now included in the paper.

4 I would expect to see a more developed discussion section. The manuscript jumps straight to the conclusion, and seems to be mostly a repetition of the results. Lacking is a discussion of what the results suggest about cultural diversity and relevance. Around line 554, the authors claim that the “abbreviated structure may offer a culturally relevant and less burdensome way to capture key mentoring competencies”; it would be good to see a discussion of how, i.e. in what ways is the new structure more culturally relevant? How will this help support marginalised and minoritised students in practice, specifically? Thank you for this critical feedback. We expanded the discussion section to address how the refined three-factor structure (Research Skill Development, Identity and Belonging, and Career Alignment) reflect unique needs at an HSI. Specifically, in regard to ‘Identity and Belonging,’ we find that the items for ‘Addressing Diversity’ do not function as a standalone factor, but instead aligns with prior research that indicates that, for Latino and other minoritized populations, culture and diversity are not separate constructs, but rather central to their identity. We also added a discussion on how the 18-item reduces the burden for faculty at minority serving institutions (MSIs).

5 I recommend the implications section as a whole is in need of further development. Around line 282 of the manuscript, the authors argue “Successful interventions in this community are likely to benefit the broader academic community, as they address the needs of both minoritized (e.g., racial/ethnic minorities) and marginalized (e.g., low socioeconomic status) populations. Therefore, interventions that prove effective in this environment can be expected to work well in other settings” – how can the results be used to help support students from diverse communities? I understand the study was a revalidation study, but a short section highlighting some recommendations for mentors could increase the impact and utility of the study. Thank you for the suggestion to increase the utility of the study for mentors. We have added recommendations with implications for mentoring. Specifically, we translate our three-factor structure of the mentoring competency scale into specific practices, such as prioritizing ‘Identity and Belonging’ through cultural humility and explicitly expanding discussion of professional development beyond career planning, but situating it in the context of socio-economic challenges faced by many students at HSIs and helping them understand topics on how to be effective role models and leveraging their networks.

6 The conclusion makes some claims that I’m not sure are fully supported by the results, as they are currently presented, e.g. “Importantly, our structure also reinforces the view that culturally responsive mentoring, acknowledging mentees' diverse backgrounds and building inclusive communication practices, for example, is not a separate domain, but rather embedded throughout mentoring relationships” (lines 556-8), and “For our final model, items tied to diversity considerations clustered within the communication and professional development factors, suggesting that equity and inclusion are foundational to effective mentorship” (lines 599-61). More developed discussion of the results is needed here to link these claims to the results of the GFA. The claims are now supported by demonstrating the integration of diversity-related items into the other core factors, and a discussion of the results to link the clustering of diversity items to the conclusion that equity and inclusion are foundational to effective mentorship

7 A minor point that the journal requires referencing in Vancouver style Thank you for this suggestion. References have now been converted to Vancouver style.

Review 2 Comments

8 1. The writing is verbose and repetitive, necessitating content reduction. For instance, the abstract should only introduce the research objectives, methods, results, and conclusions, with a focus on highlighting the author's own work. The work of other scholars can be briefly introduced as research background, rather than being extensively elaborated upon. There is no need for lengthy introductions of other scholars' work. Additionally, the extensive introduction of PCA and ICA in the "Statistical Approach" section is unnecessary. It is recommended to streamline this section and merge it with the "Comparing PCA and FA to EGA" section. Thank you for this feedback. We agree and have streamlined the manuscript for clarity. The abstract has been revised to focus strictly on our objectives, the EGA methodology, and the key finding of a three-factor structure. We significantly reduced the theoretical background of PCA and ICA, only keeping the “Comparing PCA and FA to EGA” sub-section. This allows the reader to focus on the current study's application of network-based methods rather than a long discussion of historical statistical techniques.

9 2. The overall logic of the article is unclear, and the structure is somewhat disorganized, requiring careful reorganization. For example, the paper emphasizes the application of the original Mentoring Competency Assessment in clinical and biomedical research contexts, but the relationship between this and the current study is unclear. This paper merely extends this MCA to Hispanic contexts for re-evaluation, without sufficiently analyzing the expansion of MCA's application across disciplines. Furthermore, the literature review specifically mentions that "Hyun and colleagues (2022) conducted a large-scale revalidation of the MCA using data from 1,626 mentors across a wider range of research institutions and disciplines," indicating the importance of Hyun's work. However, since this paper does not build upon Hyun's work nor compare its findings with Hyun's, it is unclear why the author devotes significant space to introducing Hyun's work. Additionally, the author does not analyze the reasons for the differences between the re-validated MCA and the original dimensions. In the methodology section, the author states that they used a 7-point Likert-type scale for the MCA, but it remains unclear whether Fleming et al.'s MCA also employed a 7-point scale. We have reorganized the literature review to better bridge the gap between previous validation studies and our own. We included Fleming (2013) and Hyun (2022) to establish the current 'Gold Standard' (6-factor model) and to highlight that even large-scale revalidations (Hyun, 2022) have relied on traditional CFA/SEM. Our study builds upon their work by testing if those same six factors hold true in a specific cultural context (HSI) using a novel methodology (EGA). We have added a comparison in the Discussion section detailing why our 3-factor structure emerged in contrast to their 6-factor structure, specifically noting that in our sample, 'Diversity' and 'Communication' items collapsed into 'Identity and Belonging.' Finally, we clarified that the 7-point Likert scale used is consistent with the original MCA developed by Fleming et al.

10 3. The methodology of this paper requires further clarification. For instance, it is uncertain whether the language of the questionnaire used in the Hispanic Serving Institutions matches the language of the research participants. I apologize for my lack of familiarity with the situation in the United States as a foreigner, and I would appreciate it if the author could clarify this point. Additionally, with only 323 valid participants in this study, the sample size is relatively small. The author should either increase the number of questions to expand the sample size or provide an explanation and declare this limitation in the research limitations section. We have clarified in the 'Participants' section that the survey was administered in English, which is the primary language of instruction and professional research at this HSI. Regarding sample size, while N=323 is smaller than some national studies, it exceeds the requirements for stable Exploratory Graph Analysis (EGA). To address the reviewer’s concern, we performed a post-hoc Monte Carlo power analysis which demonstrated that our sample size provided 100% power to detect the identified latent structure. We have also added this as a limitation regarding generalizability to non-English speaking or non-HSI contexts

11 4. Although the paper lists a substantial amount of work by other scholars in the field of MCA, the author fails to compare and analyze their own work with that of others, nor do they explain how their work critiques or builds upon the work of others. Consequently, it is difficult to assess the creativity of this study. The creativity of this study lies in the intersection of cultural context and advanced psychometrics. By applying EGA we move beyond the traditional factor analysis. Our study critiques the 6-factor model by showing that for mentors at HSIs, competencies like 'Addressing Diversity' are not siloed skills but are fundamentally integrated into 'Identity and Belonging.' This provides a more culturally nuanced and 'less burdensome' 18-item tool for future research in diverse settings.

Review 3 Comments

12 This study revalidated the MCA tool by applying EGA technology and proposed a more concise three-factor structure. This research offers a new perspective for the improvement of mentorship competency assessment tools, especially in institutions serving underrepresented minority students. However, the limitations of the study should not be overlooked, particularly in terms of sample representativeness and data collection methods. It is recommended that the authors consider the above comments in future revisions, further refine the research design and analysis methods, and enhance the reliability and generalizability of the research findings. We thank the reviewer for their positive assessment of the value of our work at Hispanic-Serving Institutions. We have taken the concerns regarding sample representativeness and data collection methods seriously. Specifically, we have:

1. Expanded the Limitations section to address the single-institution nature of the study and the use of self-reported data.

2. Added a Future Research section that outlines a roadmap for multi-institutional and longitudinal validation.

3. Provided a Post-hoc Power Analysis to support the reliability of our findings despite the smaller sample size relative to national studies

13 1. Although the study sample was drawn from a representative HSI, the sample was limited to a single institution, which may restrict the generalizability of the findings. We agree that the single-institution nature of this study is a limitation. While our institution is highly representative of HSIs in the Southwest, we have updated the Limitations section to explicitly state that the three-factor structure should be viewed as a 'context-specific model.' We also indicate in future directions that future multi-site studies to investigate if this structure holds across other HSIs and/or MSIs with different regional or demographic profiles.

14 2. The study mentions sample diversity mainly in terms of race and ethnicity but does not elaborate on other factors that may affect mentoring competencies, such as mentors' disciplinary backgrounds and career stages. It is suggested that future research further explore the impact of these variables on the mentorship competency assessment tool. This is an excellent point. While our primary focus was the cultural context of an HSI, factors such as disciplinary areas and career stage likely influence mentoring priorities. We have added this to our 'Future Directions' section, suggesting that future EGA models include these variables as covariates to see if the network structure shifts based on professional experience or discipline

15 3. The study utilized self-reported data from mentors, which may introduce social desirability or recall biases. It is recommended that future research consider incorporating assessments from mentees or independent observations to provide a more comprehensive evaluation perspective. We acknowledge that self-report data is subject to social desirability and recall bias. We have noted this in the Limitations section. We have also added a recommendation for pairing with student assessments, as well as conducting studies using a longitudinal framework.

16 4. The results indicate that the original six-factor structure is not applicable to the new sample, instead forming a three-factor structure. This finding is significant for improving the mentorship competency assessment tool, but further validation is needed to confirm the stability of this structure across different disciplines and institutional contexts. We agree. While our study successfully identified a new three-factor structure for this HSI population, we explicitly state in our limitations and future directions that this should be viewed as a 'context-specific' model, and that further validation is needed to see if this same structure holds true at other types of institutions or longitudinally.

17 5. While the study proposes directions for future research, it is suggested to more specifically indicate how interdisciplinary and inter-institutional research can further validate and refine the MCA tool. Additionally, it is recommended to explore how to incorporate mentees' perspectives into the mentorship competency assessment and how to evaluate the predictive validity of the MCA tool through longitudinal studies. Thank you for these suggestions. Our Future Research section now highlights these exact paths. We specify that the next logical steps for this work include:

1. Multi-site/Inter-institutional studies to see if the 18-item version works across different campuses.

2. Mentee perspectives, as our current study focused on mentor self-reports.

3. Longitudinal research to see if these

Attachment Submitted filename: Reviewer Comments final.docx

# Peer review: Decision Letter 1

**DOI:** 10.1371/journal.pone.0350417.r003

**Authors:** Mary Diane Clark

7 May 2026

PONE-D-25-32929R1

Assessing Undergraduate Mentoring Competency in a Research-Intensive Hispanic Serving Institution: A Revalidation

PLOS One

Dear Dr. Wagler,

Thank you for submitting your manuscript to PLOS ONE. After careful consideration, we feel that it has merit but does not fully meet PLOS ONE’s publication criteria as it currently stands. Therefore, we invite you to submit a revised version of the manuscript that addresses the points raised during the review process.

There are a few minor issues that need to be addressed. Then we can move forward towards finalizing the manuscript

Please submit your revised manuscript by Jun 21 2026 11:59PM. If you will need more time than this to complete your revisions, please reply to this message or contact the journal office at plosone@plos.org. When you're ready to submit your revision, log on to https://www.editorialmanager.com/pone/ and select the 'Submissions Needing Revision' folder to locate your manuscript file.

Please include the following items when submitting your revised manuscript:

- A letter that responds to each point raised by the academic editor and reviewer(s). You should upload this letter as a separate file labeled 'Response to Reviewers'.
- A marked-up copy of your manuscript that highlights changes made to the original version. You should upload this as a separate file labeled 'Revised Manuscript with Track Changes'.
- An unmarked version of your revised paper without tracked changes. You should upload this as a separate file labeled 'Manuscript'.

If you would like to make changes to your financial disclosure, please include your updated statement in your cover letter. Guidelines for resubmitting your figure files are available below the reviewer comments at the end of this letter.

If applicable, we recommend that you deposit your laboratory protocols in protocols.io to enhance the reproducibility of your results. Protocols.io assigns your protocol its own identifier (DOI) so that it can be cited independently in the future. For instructions see: https://journals.plos.org/plosone/s/submission-guidelines#loc-laboratory-protocols. Additionally, PLOS ONE offers an option for publishing peer-reviewed Lab Protocol articles, which describe protocols hosted on protocols.io. Read more information on sharing protocols at https://plos.org/protocols?utm_medium=editorial-email&utm_source=authorletters&utm_campaign=protocols.

As the corresponding author, your ORCID iD is verified in the submission system and will appear in the published article. PLOS supports the use of ORCID, and we encourage all coauthors to register for an ORCID iD and use it as well. Please encourage your coauthors to verify their ORCID iD within the submission system before final acceptance, as unverified ORCID iDs will not appear in the published article. *Only*  the individual author can complete the verification step; PLOS staff *cannot*  verify ORCID iDs on behalf of authors.

We look forward to receiving your revised manuscript.

Kind regards,

Mary Diane Clark, PhD

Academic Editor

PLOS One

Journal Requirements:

1. If the reviewer comments include a recommendation to cite specific previously published works, please review and evaluate these publications to determine whether they are relevant and should be cited. There is no requirement to cite these works unless the editor has indicated otherwise.

2. Please review your reference list to ensure that it is complete and correct. If you have cited papers that have been retracted, please include the rationale for doing so in the manuscript text, or remove these references and replace them with relevant current references. Any changes to the reference list should be mentioned in the rebuttal letter that accompanies your revised manuscript. If you need to cite a retracted article, indicate the article’s retracted status in the References list and also include a citation and full reference for the retraction notice.

Additional Editor Comments:

The manuscript is much improved. Thank you for the care you took in responding to reviewers comments.

As you may or may not know, PLOS ONE does not have a copy editor, so I must ask you to fix some things I have identified within the manuscript. Please see the attached file for these comments.

When you address those, I will then be able to recommend accepting the manuscript

[Note: HTML markup is below. Please do not edit.]

Reviewers' comments:

Reviewer's Responses to Questions

**Comments to the Author**

1. If the authors have adequately addressed your comments raised in a previous round of review and you feel that this manuscript is now acceptable for publication, you may indicate that here to bypass the “Comments to the Author” section, enter your conflict of interest statement in the “Confidential to Editor” section, and submit your "Accept" recommendation.

Reviewer #6: All comments have been addressed

**********

2. Is the manuscript technically sound, and do the data support the conclusions?

The manuscript must describe a technically sound piece of scientific research with data that supports the conclusions. Experiments must have been conducted rigorously, with appropriate controls, replication, and sample sizes. The conclusions must be drawn appropriately based on the data presented.

Reviewer #6: Yes

**********

3. Has the statistical analysis been performed appropriately and rigorously?

Reviewer #6: Yes

**********

4. Have the authors made all data underlying the findings in their manuscript fully available?

The PLOS Data policy requires authors to make all data underlying the findings described in their manuscript fully available without restriction, with rare exception (please refer to the Data Availability Statement in the manuscript PDF file). The data should be provided as part of the manuscript or its supporting information, or deposited to a public repository. For example, in addition to summary statistics, the data points behind means, medians and variance measures should be available. If there are restrictions on publicly sharing data—e.g. participant privacy or use of data from a third party—those must be specified.

Reviewer #6: Yes

**********

5. Is the manuscript presented in an intelligible fashion and written in standard English?

PLOS ONE does not copyedit accepted manuscripts, so the language in submitted articles must be clear, correct, and unambiguous. Any typographical or grammatical errors should be corrected at revision, so please note any specific errors here.

Reviewer #6: Yes

**********

6. Review Comments to the Author

Please use the space provided to explain your answers to the questions above. You may also include additional comments for the author, including concerns about dual publication, research ethics, or publication ethics. (Please upload your review as an attachment if it exceeds 20,000 characters)

Reviewer #6: (No Response)

**********

7. PLOS authors have the option to publish the peer review history of their article (what does this mean?). If published, this will include your full peer review and any attached files.

If you choose “no”, your identity will remain anonymous but your review may still be made public.

**Do you want your identity to be public for this peer review?**  For information about this choice, including consent withdrawal, please see our Privacy Policy.

Reviewer #6: No

**********

[NOTE: If reviewer comments were submitted as an attachment file, they will be attached to this email and accessible via the submission site. Please log into your account, locate the manuscript record, and check for the action link "View Attachments". If this link does not appear, there are no attachment files.]

To ensure your figures meet our technical requirements, please review our figure guidelines: https://journals.plos.org/plosone/s/figures

You may also use PLOS’s free figure tool, NAAS, to help you prepare publication quality figures: https://journals.plos.org/plosone/s/figures#loc-tools-for-figure-preparation.

NAAS will assess whether your figures meet our technical requirements by comparing each figure against our figure specifications.

Attachment Submitted filename: Mentoring at a HSI.docx

# Author comment: Author response to Decision Letter 2

**DOI:** 10.1371/journal.pone.0350417.r004

12 May 2026

No data in abstract

Thank you, the citation about the final model CLI is now added.

Do not use PWI----it is used a few times but often you spell it out and then abbreviate it again. Simply use the original wrding

Thank you again, This is now corrected in the manuscript.

Lit review is integrated into the first part—do not repeat it again

Thank you for this-now fully integrated.

One of the most comprehensive tools, the MCA, was developed by given this sentence frame you need the authors name here[19]-----or rephrase this sentence is a better solution. It worked before the numbers but not now

This sentence is now corrected in the manuscript.

Indent 5 spaces all paragraphs

All paragraphs are now indented. Thank you.

This below is not adding to your argument:

Effective mentoring is crucial for the development of research competencies in early career researchers [19]. Mentors provide guidance, support, and knowledge to mentees. High-quality mentoring relationships are associated with better academic and career outcomes for mentees [20][21].

I suggest deleting the whole thing

Thank you, this is now deleted as are references 20 and 21 given they were only cited here.

Add citations for these : The Walktrap, Leiden, and Louvain algorithms.

Thank you-this is now cited.

Which was the sample size—they do not agree in the data analytic vs results sections

final analytical sample size of N = 323 OR The final analyzed sample consisted of N = 363 mentors

This is corrected. The 323 was the final sample size and 363 was the size before item-wise deletion. The table figures now corrected and this is clarified in the paper.

I don’t see a description of this: modified parallel analysis

Thank you- this is now corrected and a description is added to methods.

Problem with this sentence:

A post-hoc power analysis conducted for this model using Monte Carlo simulation.

Thank you- this is now corrected with an appropriate verb added to the sentence.

Attachment Submitted filename: Response to Reviewers.docx

# Editor report: Decision Letter 2

**DOI:** 10.1371/journal.pone.0350417.r005

**Authors:** Mary Diane Clark

14 May 2026

Assessing Undergraduate Mentoring Competency in a Research-Intensive Hispanic Serving Institution: A Revalidation

PONE-D-25-32929R2

Dear Dr. Wagler,

We’re pleased to inform you that your manuscript has been judged scientifically suitable for publication and will be formally accepted for publication once it meets all outstanding technical requirements.

Within one week, you’ll receive an e-mail detailing the required amendments. When these have been addressed, you’ll receive a formal acceptance letter and your manuscript will be scheduled for publication.

An invoice will be generated when your article is formally accepted. Please note, if your institution has a publishing partnership with PLOS and your article meets the relevant criteria, all or part of your publication costs will be covered. Please make sure your user information is up-to-date by logging into Editorial Manager at Editorial Manager® and clicking the ‘Update My Information' link at the top of the page. For questions related to billing, please contact billing support.

If your institution or institutions have a press office, please notify them about your upcoming paper to help maximize its impact. If they’ll be preparing press materials, please inform our press team as soon as possible -- no later than 48 hours after receiving the formal acceptance. Your manuscript will remain under strict press embargo until 2 pm Eastern Time on the date of publication. For more information, please contact onepress@plos.org.

Kind regards,

Mary Diane Clark, PhD

Academic Editor

PLOS One

Additional Editor Comments (optional):

Thank you for fixing those few minor issues. The instrument should be extemely helpful to HSIs.

Reviewers' comments:

# Editor report: Acceptance letter

**DOI:** 10.1371/journal.pone.0350417.r006

**Authors:** Mary Diane Clark

PONE-D-25-32929R2

PLOS One

Dear Dr. Wagler,

I'm pleased to inform you that your manuscript has been deemed suitable for publication in PLOS One. Congratulations! Your manuscript is now being handed over to our production team.

At this stage, our production department will prepare your paper for publication. This includes ensuring the following:

* All references, tables, and figures are properly cited

* All relevant supporting information is included in the manuscript submission,

* There are no issues that prevent the paper from being properly typeset

You will receive further instructions from the production team, including instructions on how to review your proof when it is ready. Please keep in mind that we are working through a large volume of accepted articles, so please give us a few days to review your paper and let you know the next and final steps.

Lastly, if your institution or institutions have a press office, please let them know about your upcoming paper now to help maximize its impact. If they'll be preparing press materials, please inform our press team within the next 48 hours. Your manuscript will remain under strict press embargo until 2 pm Eastern Time on the date of publication. For more information, please contact onepress@plos.org.

You will receive an invoice from PLOS for your publication fee after your manuscript has reached the completed accept phase. If you receive an email requesting payment before acceptance or for any other service, this may be a phishing scheme. Learn how to identify phishing emails and protect your accounts at https://explore.plos.org/phishing.

If we can help with anything else, please email us at customercare@plos.org.

Thank you for submitting your work to PLOS ONE and supporting open access.

Kind regards,

PLOS ONE Editorial Office Staff

on behalf of

Dr. Mary Diane Clark

Academic Editor

PLOS One
